# Functional Divergence of *APETALA1* and *FRUITFULL* is due to Changes in both Regulation and Coding Sequence

**DOI:** 10.3389/fpls.2015.01076

**Published:** 2015-12-02

**Authors:** Elizabeth W. McCarthy, Abeer Mohamed, Amy Litt

**Affiliations:** ^1^Department of Botany and Plant Sciences, University of California, RiversideRiverside, CA, USA; ^2^Department of Agricultural Botany, Faculty of Agriculture (Saba Basha), Alexandria UniversityAlexandria, Egypt

**Keywords:** gene duplication, functional divergence, *APETALA1*, *FRUITFULL*, MADS box genes, conserved protein motifs

## Abstract

Gene duplications are prevalent in plants, and functional divergence subsequent to duplication may be linked with the occurrence of novel phenotypes in plant evolution. Here, we examine the functional divergence of *Arabidopsis thaliana APETALA1* (*AP1*) and *FRUITFULL* (*FUL*), which arose via a duplication correlated with the origin of the core eudicots. Both *AP1* and *FUL* play a role in floral meristem identity, but *AP1* is required for the formation of sepals and petals whereas *FUL* is involved in cauline leaf and fruit development. *AP1* and *FUL* are expressed in mutually exclusive domains but also differ in sequence, with unique conserved motifs in the C-terminal domains of the proteins that suggest functional differentiation. To determine whether the functional divergence of *AP1* and *FUL* is due to changes in regulation or changes in coding sequence, we performed promoter swap experiments, in which *FUL* was expressed in the *AP1* domain in the *ap1* mutant and vice versa. Our results show that *FUL* can partially substitute for *AP1*, and *AP1* can partially substitute for *FUL*; thus, the functional divergence between *AP1* and *FUL* is due to changes in both regulation and coding sequence. We also mutated AP1 and FUL conserved motifs to determine if they are required for protein function and tested the ability of these mutated proteins to interact in yeast with known partners. We found that these motifs appear to play at best a minor role in protein function and dimerization capability, despite being strongly conserved. Our results suggest that the functional differentiation of these two paralogous key transcriptional regulators involves both differences in regulation and in sequence; however, sequence changes in the form of unique conserved motifs do not explain the differences observed.

## Introduction

Gene duplications are prevalent in angiosperms, occurring via either whole genome or tandem duplications. Duplications can increase robustness of developmental processes through redundancy (Wagner, 2008) or lead to the evolution of novel or partitioned functions between duplicates through the process of neo- or subfunctionalization (Ohno, 1970; Force et al., 1999; Lynch and Force, 2000). The increase in morphological complexity observed during the evolution of plants and animals is thought to be linked to functional divergence of gene duplicates (Ohno, 1970; Freeling and Thomas, 2006). MADS box transcription factors play key roles in the gene networks directing the floral transition and floral and fruit development and through duplication have diversified dramatically in plants, particularly in seed plants (Theissen et al., 2000; Kaufmann et al., 2005). The retention of gene duplicates drove this increase in gene number, and the diversity in function that occurred subsequent to duplication contributed to the development of the complex reproductive structures that are unique to these plant lineages (Theissen et al., 2000; Kaufmann et al., 2005).

One MADS-box gene subfamily that arose via duplications is the angiosperm-specific *AP1/FUL* lineage, the members of which play key roles in several important developmental processes including flower and fruit development. Multiple duplications have occurred in this lineage, including a key event that coincided with the origin of the core eudicots; this duplication produced the *euAP1* (including *Arabidopsis AP1*) and *euFUL* (including *Arabidopsis FUL*) clades (Litt and Irish, 2003). This duplication is likely part of the whole genome triplication that occurred before the diversification of the core eudicots, often referred to as the gamma event (Jiao et al., 2012). In *Arabidopsis*, *AP1* is required for proper specification of floral meristem identity and for sepal and petal development; in strong *ap1* mutants, petals are not formed and sepals are transformed into bract-like organs (Irish and Sussex, 1990; Bowman et al., 1993). Secondary flowers can grow from the axils of these first whorl organs, and reiterate the phenotype, so that tertiary and quaternary flowers can occur (Irish and Sussex, 1990), indicating partial retention of inflorescence identity. In *Arabidopsis ful* mutants, fruit development is disrupted. Cells in the valves of the silique fail to elongate and differentiate, resulting in a short fruit; seed development proceeds as normal, which leads to over-crowding and premature rupture of the fruit wall (Gu et al., 1998). In addition, the cauline leaves of *ful* mutants are wider than those of wild type (WT; Gu et al., 1998). AP1 excludes *FUL* from the floral meristem (Mandel and Yanofsky, 1995a), and the single *ful* mutant shows no defects in flower development (Gu et al., 1998). However, in *ap1-1* mutants, *FUL* is ectopically expressed in the floral meristem and further loss of floral identity is observed when *FUL* function is lost as well, indicating that *FUL* also is capable of specifying floral meristem identity; however, it cannot fully compensate for loss of *AP1* function (Ferrandiz et al., 2000). Thus, *AP1* and *FUL* are redundant for one function, floral meristem identity, but otherwise have diverged functionally, playing distinct roles in perianth identity and in cauline leaf and fruit development, respectively.

Although their sequences are similar, as is expected from paralogs, the AP1 and FUL proteins have differing conserved motifs in their C-terminal domains. All FUL proteins, as well as the related SEPALLATA (SEP) and AGAMOUS-LIKE 6 (AGL6) proteins, have a six hydrophobic amino acid motif (LPAWML), the FUL-like motif, near the C terminus (Litt and Irish, 2003; Zahn et al., 2005; Shan et al., 2007). The function of this motif is unknown. AP1 has lost this FUL-like motif, due to a single nucleotide frame shift in the 3′ end of the coding sequence (Litt and Irish, 2003; Vandenbussche et al., 2003). Instead AP1 proteins have a transcription activation domain (Cho et al., 1999) and terminate in a farnesylation (a type of prenylation) domain (Yalovsky et al., 2000; Litt and Irish, 2003). The farnesylation domain directs the addition of a lipid moiety to the C terminus of the protein, and AP1 proteins have been shown to be farnesylated *in vivo* (Yalovsky et al., 2000). Farnesylation is often implicated in targeting proteins to membranes, and can be a mechanism for regulating transcription factor activity (Resh, 2006). Alternatively, it has been suggested that this motif may mediate protein interactions (Yalovsky et al., 2000). Overexpression of a mutated version of the AP1 protein, in which farnesylation was abolished, in WT *Arabidopsis* did not completely recapitulate the *AP1* overexpression phenotype and additionally displayed novel phenotypes, suggesting a role for this protein modification in AP1 function (Yalovsky et al., 2000). However, overexpression of related proteins which lack the farnesylation motif can also induce a phenotype similar to that of *AP1* overexpression (Berbel et al., 2001; Blázquez et al., 2001; Castillejo et al., 2005; Chen et al., 2008). Thus the importance of these motifs to protein function is not clear; however, the presence of these sequence differences in the C-terminal protein domains, as well as other sequence differences between the proteins, is one possible explanation for the differences in function of AP1 and FUL.

A second possible explanation is that *AP1* and *FUL* are expressed in mutually exclusive domains in *Arabidopsis*. *AP1* is first expressed throughout young stages 1 and 2 floral meristems, but by stage 3 expression is restricted to the periphery of the floral meristem, where first and second whorl organs will arise (Mandel et al., 1992). *AP1* expression is maintained in sepal and petal primordia as flowers develop (Mandel et al., 1992). In contrast, *FUL* expression is first seen in the inflorescence meristem at the onset of reproductive development and is found in the inflorescence meristem, the stem, and cauline leaves as inflorescence development continues (Mandel and Yanofsky, 1995a). *FUL* is not expressed in the floral meristem until stage 3, at which time it is found in the central dome, where the fourth whorl organs will arise; in later floral development, *FUL* is expressed in the valves of the developing carpels (Mandel and Yanofsky, 1995a). Thus, there is no overlap in the WT expression patterns of *AP1* and *FUL*, at least partly because AP1 represses *FUL* expression (Mandel and Yanofsky, 1995a), presenting another possible explanation for the functional differences seen between AP1 and FUL.

Pre-duplication *FUL*-like genes show broad expression patterns, with transcript generally present in the shoot apical meristem, leaves, inflorescence and floral meristems, and in most if not all floral organs and fruits (e.g., Yu and Goh, 2000; Pelucchi et al., 2002; Murai et al., 2003; Tsaftaris et al., 2004; Kim et al., 2005; Li et al., 2005; Preston and Kellogg, 2006; Pabón-Mora et al., 2012, 2013; Acri-Nunes-Miranda and Mondragón-Palomino, 2014; Sun et al., 2014). Basal eudicots belong to the same clade as the core eudicots, but diverged prior to the duplication that created the *euAP1* and *euFUL* clades. *FUL*-like genes in these species are involved in regulation of flowering time, inflorescence branching, and cauline leaf, sepal, petal, carpel, and fruit development, essentially encompassing all the functions of *euAP1* and *euFUL* genes combined (Pabón-Mora et al., 2012; however, see Pabón-Mora et al., 2013). This suggests that subfunctionalization and partitioning of the ancestral *FUL*-like functions among the *euAP1* and *euFUL* genes occurred following the core eudicot duplication. These data from *FUL*-like genes set the stage for examination of the post-duplication evolutionary patterns of *euAP1* and *euFUL* gene lineages.

Here, we examine the basis of functional differentiation between *AP1* and *FUL* in *Arabidopsis thaliana* to determine if observed differences are due to their mutually exclusive expression domains or differences in their protein sequences. We perform promoter swap experiments and also investigate the role of conserved motifs in protein function through site-directed mutagenesis. In addition, because MADS-domain proteins act in complexes, we evaluated the ability of the mutated proteins to bind with known AP1 and FUL MADS-domain protein interacting partners. Our results suggest that the functional divergence of *AP1* and *FUL* is due to changes in both regulation and coding sequence, and that the conserved motifs of AP1 and FUL may not play major roles in protein function.

## Materials and Methods

### Plant Material and Growth Conditions

The following mutant lines were obtained from The Arabidopsis Information Resource (TAIR): CS28, *ap1-1* mutant in a L*er* background; CS3759, *ful-1* mutant in a L*er* background. The *ap1-1* mutation (hereafter referred to as *ap1*) is a strong allele in which there is a mutation in the splice acceptor site of the third intron, resulting in transcript in which the third intron is retained (Mandel et al., 1992). The *ful-1* mutation (hereafter referred to as *ful*) was produced via transposon-mediated enhancer trap mutagenesis, and the insertion of a DsE element into the 5′ UTR of the *FUL* gene yields a null mutation (Gu et al., 1998). WT CS20 L*er* seeds were kindly provided by Michael Purugganan (New York University, New York, NY, USA). These lines were grown under 16 h light and 8 h dark at 21°C and 60% humidity.

### Cloning of *AP1* and *FUL* Genes

To clone the *AP1* and *FUL* promoters, genomic DNA was extracted from WT CS20 L*er* plants using phenol:chloroform (see Supplemental Materials and Methods for details). The 1.7 kilobase (kb) *AP1* promoter (Hempel et al., 1997; Alvarez-Buylla et al., 2006) was amplified using primers GL373F and GL374R, cloned into pCR 2.1-TOPO (Invitrogen), and sequenced in both directions. For the *FUL* promoter, we used a 4.2 kb fragment upstream of the *FUL* coding sequence that includes all of the identified upstream regulatory sequence (Nguyen, 2008; Woods, 2010). The *FUL* promoter was amplified using primers AN19F and AN20R, cloned into pCR XL-TOPO, and sequenced in both directions. A second amplification was performed to add a SacI restriction site to the 5′ end of the *FUL* promoter (primers GL395F and AN20R); this product was also cloned into pCR XL-TOPO and sequenced in both directions. Promoter sequences used also contained the 5′ UTR of the mRNA. Primer sequences are found in Supplementary Table S1, and PCR conditions are found in Supplemental Materials and Methods.

To clone the *AP1* and *FUL* coding sequences, RNA was extracted from inflorescence tissue of WT CS20 L*er* plants using the RNeasy Plant Mini Kit (Qiagen) and DNase treated using the TURBO DNase-free kit (Ambion), according to manufacturers’ instructions. Two micrograms of RNA were reverse transcribed into cDNA using SuperScript III (Invitrogen) according to the manufacturer’s protocol. The *AP1* coding sequence with the 3′ UTR was amplified using primers GL1090F and GL1091R, cloned into pCR 2.1-TOPO, and sequenced in both directions. The *FUL* coding sequence with the 3′ UTR was amplified using primers GL314F and GL315R, cloned into pCR 4-TOPO, and sequenced in both directions. Coding sequences used began with the start codon and included the 3′ UTR.

### Cloning of Constructs

Chimeric PCR was performed to link the promoters and coding sequences and, with site-directed mutagenesis, to produce mutated coding sequences. Chimeric PCR consists of three PCR reactions, the first of which (PCR1) amplifies the 5′ portion of the final product, and the second of which (PCR2) amplifies the 3′ portion; these two products contain complementary and overlapping sequence. The third PCR (PCR3) uses both of these products as template, and amplification links them together into a single product. To link promoters and coding sequences, the first PCR used a forward primer that anneals to the 5′ end of the promoter and a reverse primer that binds to the 3′ end of the promoter, but also includes sequence complementary to the beginning of the coding sequence (Supplementary Figure S1A). The second PCR used a forward primer that binds to the 5′ end of the coding sequence and that includes sequence complementary to the 3′ end of the promoter. The reverse primer binds to the 3′ end of the coding sequence (Supplementary Figure S1A). The third PCR used the products of the first two PCRs as mixed templates and primers that bind to the 5′ end of the promoter and the 3′ end of the coding sequence. Because of the design of the internal primers (the reverse primer from PCR1 and the forward primer from PCR2), the products from the first two PCRs were complementary across the promoter/coding sequence link and annealed together; extension and amplification resulted in a seamless link between promoter and coding sequence (Supplementary Figure S1A).

Site-directed mutagenesis and chimeric PCR were also used to create mutated coding sequences. In this case, the internal primers were complementary and annealed across the target motif (Supplementary Figure S1B). The primers themselves included base pair mismatches that introduced point mutations to create the mutated coding sequences required. The third PCR used primers that amplified the entire coding sequence.

### Promoter Swap Constructs

**FIGURE 1:**
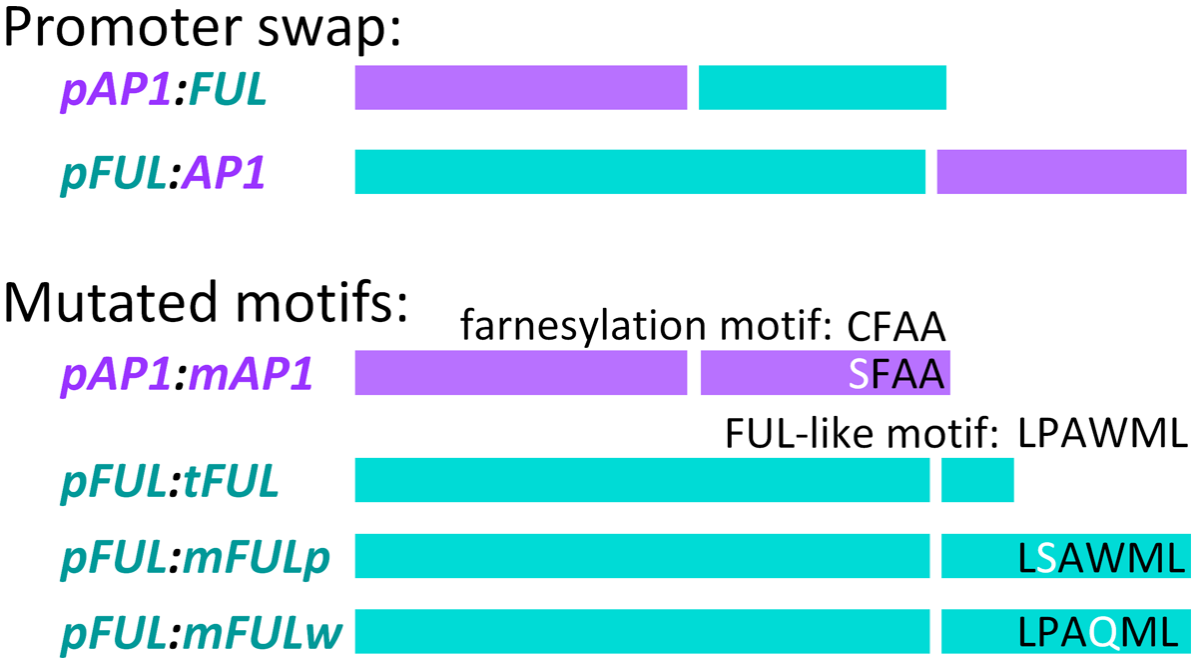
**Promoter swap and mutated motif constructs**. In each construct, the left-hand of the two bars represents the promoter (color-coded, purple for AP1 and blue for FUL) and the right hand bar represents the coding sequence (similarly color coded) Descriptions of the constructs are in the text.

In order to examine whether *FUL* can substitute for *AP1* function when expressed in the *AP1* domain and vice versa, we created the promoter swap constructs, *pAP1:FUL* and *pFUL:AP1* along with the positive control constructs, *pAP1:AP1* and *pFUL:FUL* (**Figure 1**). We performed chimeric PCRs in which PCR1 amplified the promoter, PCR2 amplified the coding sequence, and PCR3 linked them (see above for description of chimeric PCR). The *pAP1:FUL* construct was produced using primers AN21F and AN22R for PCR1, AN24F and GL315R for PCR2, and GL373F and GL315R for PCR3, whereas the *pAP1:AP1* construct was created using primers AN21F and AN23R for PCR1, AN25F and GL1419R for PCR2, and AN21F and AN15R for PCR3. These full length promoter:coding sequence constructs (PCR3 products) were cloned into pCR TOPO-2.1, and sequenced in both directions.

In order to prevent the introduction of errors via unnecessary reamplification of the long, previously cloned *FUL* promoter, we used a combination of chimeric PCR and restriction digests in the creation of the *pFUL:AP1* and *pFUL:FUL* constructs. Chimeric PCR was performed to link the *AP1* and *FUL* coding sequences with a 200 bp fragment of the 3′ end of the *FUL* promoter, using, for *pFUL:AP1*, primers AN10F and AN12R for PCR1, AN14F and AN15R for PCR2, and AN10F and AN15R for PCR3, and for *pFUL:FUL*, primers AN10F and AN11R for PCR1, AN13F and GL315R for PCR2, and AN10F and GL315R for PCR3. The 200 bp fragment of the promoter includes a naturally occurring AccI restriction site, and the reverse primer in PCR3 introduced a BamHI restriction site at the end of the 3′ UTRs. PCR3 products, which consisted of 200 bp of the promoter and the coding sequence with the BamHI site, were cloned into pCR 2.1-TOPO and sequenced in both directions. Restriction digest with AccI [New England Biolabs (NEBs)] and BamHI (NEB) was performed on both the full-length *FUL* promoter clone in the pCR TOPO-XL vector and on PCR3 clones. Fragments of the appropriate length were excised from an agarose gel, purified using the QIAEX II Gel Extraction Kit (Qiagen), ligated using T4 DNA ligase (NEB), and sequenced in both directions.

### Mutated Protein Constructs

Site-directed mutagenesis and chimeric PCR were used to create mutated coding sequences in order to examine the role of conserved motifs in protein function. To abolish the AP1 farnesylation motif, following Yalovsky et al. (2000), the receptor cysteine was replaced by serine to create the *mAP1* mutated coding sequence (**Figure 1**). Reactions used primers GL1090F and AN186R to amplify from the start codon to the farnesylation motif (PCR1), AN185F and AN15R to amplify from the farnesylation motif to the end of the 3′ UTR (PCR2), and GL1090F and AN15R to amplify the entire mutated coding sequence (PCR3).

To evaluate the function of the FUL-like motif, we constructed three mutated coding sequences (**Figure 1**). In all three cases, PCR1 amplified from the start codon to the FUL-like motif, PCR2 amplified from the FUL-like motif to the end of the 3′ UTR, and PCR3 amplified the entire mutated coding sequence. To produce the *tFUL* mutated coding sequence, a stop codon was introduced just before the FUL-like motif, truncating the FUL protein and eliminating the motif; *tFUL* was produced using primers GL314F and GL769R for PCR1, GL768F and GL315R for PCR2, and GL314F and GL315R for PCR3. To generate the *mFULp* mutated coding sequence, which resulted in a protein in which the non-polar proline of the FUL-like motif (LPAWML) was replaced with a polar serine, we used primers GL10F and GL1238R for PCR1, GL1237F and GL315R for PCR2, and GL314F and GL315R for PCR3. To create the *mFULw* mutated coding sequence, which resulted in a protein in which the non-polar tryptophan of the FUL-like motif was replaced with a polar glutamine, we used primers GL10F and GL1236R for PCR1, GL1235F and GL315R for PCR2, and GL314F and GL315R for PCR3.

Chimeric PCR was used to link the *AP1* and *FUL* promoters to the various mutated coding sequences. PCR1 amplified the promoter, PCR2 amplified the coding sequence, and PCR3 linked the promoter and coding sequence. The *pAP1:mAP1* construct was created using primers AN21F and AN23R for PCR1, AN25F and GL1419R for PCR2, and AN21F and AN15R for PCR3. Products from PCR3 were cloned into pCR TOPO-2.1 and sequenced in both directions.

For the *pFUL:tFUL*, *pFUL:mFULp*, and *pFUL:mFULw* constructs, primers AN10F and AN11R were used for PCR1, AN13F and GL315R for PCR2, and AN10F and GL315R for PCR3. The PCR3 product for each mutated coding sequence was cloned into pCR TOPO-2.1 and sequenced in both directions. Restriction digests, gel excision and purification, ligation, and sequencing were performed as described above.

### Cloning into Binary Vectors

All constructs were reamplified to introduce the sequence CACC at the 5′ end of the promoter for directional cloning. *pAP1:AP1* and *pAP1:mAP1* were amplified using primers AN305F and AN15R; *pAP1:FUL* was amplified using primers AN305F and AN306R; *pFUL:FUL*, *pFUL:tFUL*, *pFUL:mFULp*, and *pFUL:mFULw* were amplified using primers AN303F and GL315R; and *pFUL:AP1* was amplified using primers AN304F and GL1419R. The resulting PCR products were cloned into pENTR/D-TOPO (Invitrogen) and sequenced. Constructs with the *AP1* promoter were recombined into the Gateway pK7WG binary vector, and those with the *FUL* promoter were recombined into the pH7WG binary vector (Department of Plant Systems Biology, Ghent University, Belgium) using LR Clonase II (Invitrogen). Empty pK7WG and pH7WG vectors were created by recombination with an empty pENTR/D-TOPO vector in order to remove the *ccd*B screening gene. Each construct was transformed into *Agrobacterium tumefaciens* strain GV3101 (pMP90).

### Plant Transformation

Floral dip transformation with *Agrobacterium* was performed following Clough and Bent (1998). Briefly, *Agrobacterium* cultures were resuspended in infiltration medium (0.5x Murashige and Skoog salts with Gamborg’s vitamins, 5% sucrose, 0.05% MES, 0.044 μM benzylaminopurine, 0.02% Silwet L-77) to OD_600_ = 0.8. Plants (*ap1* mutants for constructs using the *AP1* promoter and *ful* mutants for constructs using *FUL* promoter) were dipped into the *Agrobacterium* solution for 15 min and then pots were placed on their sides, covered to maintain humidity, and left in the dark overnight. Pots were placed upright, returned to the growth chamber (16 h light and 8 h dark, 21°C, 60% humidity), and left to grow until siliques matured. Seed was collected and screened for transformants.

### Transformant Screening

T_1_ and T_2_ seed from all constructs was screened on MS plates (1x Murashige and Skoog with Gamborg’s vitamins, 1% sucrose, 0.05% MES, 0.8% agar) with the appropriate antibiotic: 50 μg/mL kanamycin for pK7WG constructs and 15 μg/mL hygromycin for pH7WG constructs (hygromycin plates had no sucrose). Seedlings on plates were grown under 16 h light and 8 h dark at 21°C and 60% humidity, and seedlings that grew true leaves were deemed putative transformants and transferred to soil.

RNA was extracted from putative transformants from inflorescence tissue for *ap1* mutants and from inflorescence and silique tissue for *ful* mutants, and cDNA was synthesized as described above. The resulting cDNA was screened for presence of transgene insertion using PCR for kanamycin, using primers GL418F and GL419F, or hygromycin, using primers AN210F and AN211R, and for expression of the construct using the following strategies.

The *ap1-1* mutant has a point mutation that disrupts the splice acceptor site for the third intron, but transcript is still expressed (Mandel et al., 1992). Therefore, we designed screening primers (primers AN334F and AN215R) that amplify only the WT *AP1* transcript from our *pAP1:AP1* and *pAP1:mAP1* constructs, but not the endogenous transcript from the *ap1* mutant (see Results for further information). Similarly, to distinguish between expression of *FUL* transcript from our *pAP1:FUL* construct and endogenous *FUL* mRNA in *ap1* mutants, we designed a forward primer in the *AP1* 5′ UTR and a reverse primer in the *FUL* coding sequence (primers AN299F and AN301R).

The *ful-1* mutation is an insertion of a DsE transposable enhancer trap element in the 5′ UTR of the *FUL* gene, and no mRNA transcript is produced (Gu et al., 1998). However, to be certain that we were only amplifying expression from our constructs, we designed a forward primer (AN379F) that includes sequence on either side of the DsE insertion site in order to screen for expression in the *ful-1* mutant background. To screen for the expression of the *pFUL:FUL*, *pFUL:tFUL, pFUL:mFULp*, and *pFUL:mFULw* constructs in putative transformants, we used primers AN379F and AN380R. To screen for the expression of the *pFUL:AP1* construct, we used primers AN379F and AN381R. To distinguish between transcripts expressed from *pFUL:FUL*, *pFUL:tFUL*, *pFUL:mFULp*, and *pFUL:mFULw* constructs, we designed additional reverse primers across the engineered point mutations in the FUL-like motif: AN383R for *tFUL*, AN385R for *mFULp*, and AN386R for *mFULw*, all used with forward primer AN382F. We used actin as a control (primers AN221F and AN222R). Primer sequences are found in Supplementary Table S1, and PCR conditions are found in Supplemental Materials and Methods.

### Scoring Transformant Phenotypes

Both T_1_ and T_2_ transformants were scored for phenotypes; results are reported for T_2_ plants. For experiments that tested the ability of a construct to complement the *ap1* mutant (*pAP1:FUL ap1* and *pAP1:mAP1 ap1* lines), flowers 1–5 and 11–15 were scored for the number of flowers per pedicel. In addition, the number of petals per flower and the identity of first and second whorl organs were recorded for both primary and secondary flowers for flowers 1–5 and 11–15. For experiments that tested the ability of a construct to complement the *ful* mutant (*pFUL:AP1 ful, pFUL:tFUL ful*, *pFUL:mFULp ful*, and *pFUL:mFULw ful* lines), cauline leaf length and width were scored for the primary inflorescence and flowers 1–15 were scored for silique length. ANOVAs and Tukey’s Honest Significant Difference tests were performed in RStudio version 0.98.490, and Bonferroni corrections were applied to all statistical tests conducted.

### Yeast Two-hybrid Analysis

Yeast two-hybrid analyses were performed to determine whether the mutated AP1 and FUL coding sequences created here could still bind with known MADS-domain AP1 and FUL interaction partners. Yeast two-hybrid vectors were obtained from TAIR (www.arabidopsis.com) for AGAMOUS-LIKE 24 (AGL24), AP1, APETALA3 (AP3), FUL, PISTILLATA (PI), SEP1, and SUPPRESSOR OF CONSTANS1 (SOC1) and created for mAP1, tFUL, mFULp, mFULw, AGAMOUS (AG), AGL6, SEP3, SEP4-II, and SHORT VEGETATIVE PHASE (SVP). We amplified full-length coding sequences using the following primer combinations: AG (primers AN237F and AN238R), AGL6 (primers AN235F and AN373R), SEP3 (primers AN233F and AN234R), SEP4-II (primers AN241F and AN242R), and SVP (primers AN239F and AN240R). We amplified the full-length coding sequences for *tFUL*, *mFULp*, and *mFULw* using primers AN246F and AN247R. AP1 has been reported to have autoactivation capability (Pelaz et al., 2001), so we used chimeric PCR to create additional vectors (designated AP1-PGA and mAP1-PGA) for both WT AP1 and mAP1 in which the proline- and glutamine-rich regions and the activation domain were removed from the C-terminal region of the protein (Supplementary Figure S2A). We used this approach instead of protein truncation because we were evaluating whether the farnesylation motif at the C terminus of the protein is necessary for protein–protein interactions. We performed chimeric PCR in which PCR1 amplified from the start codon until just before the proline-rich domain, PCR2 amplified from just after the activation domain to the stop codon, and PCR3 linked these two regions, creating a coding sequence from which the proline- and glutamine-rich regions and the activation domain were removed. For AP1-PGA, we used the following primers: AN243F and AN248R (PCR1), AN249F and AN244R (PCR2), and AN243F and AN244R (PCR3). For mAP1-PGA, we used the following primers: AN243F and AN248R (PCR1), AN249F and AN245R (PCR2), and AN243F and AN245R (PCR3). These vectors were no longer capable of autoactivation (Supplementary Figure S2B). Full-length coding sequences were cloned into pENTR/D-TOPO, sequenced in both directions, and recombined into both pDEST-AD and pDEST-DB vectors, kindly provided by David Hall (Dana-Farber Cancer Institute, Boston, MA, USA), using LR Clonase II (Invitrogen). Interactions were tested by co-transforming pairs of vectors into yeast strain AH109. AP1, mAP1, AP1-PGA, mAP1-PGA, FUL, tFUL, mFULp, and mFULw were each tested against a panel of interactors: AG, AGL6, AGL24, AP3, PI, SEP1, SEP3, SEP4-II, SOC1, and SVP in both pDEST-AD and pDEST-DB vectors. Empty pDEST-AD and pDEST-DB vectors were added to the panel as negative controls. Co-transformed yeast was plated onto selective plates (synthetic drop-out; -HWL with 0, 2.5, 5, 10, 20, and 30 mM 3AT, -AWL, and -AHWL) and growth was monitored after 3 and 6 days. The interaction patterns of the mutated proteins were compared to those of their WT counterparts to determine whether the mutation disrupted normal protein–protein interactions.

## Results

### The *ap1-1* Mutant Produces Multiple Transcripts

In developing a strategy to screen for expression of the *pAP1:AP1* and *pAP1:mAP1* constructs in the *ap1-1* mutant background, we discovered that the *ap1* transcript pool is variable in this mutant. The *ap1-1* mutant has a point mutation in the splice acceptor site of the third intron and is predicted to produce a longer fragment that includes the third intron. We originally designed screening primers across the third and fourth exon boundary, but amplification yielded a ‘wild type’ sized fragment as well as the expected longer one. Sequencing established that transcripts of three different lengths are expressed. These included a long transcript (1118 bp) in which the third intron is retained, a short transcript (925 bp) in which the third intron, fourth exon, and fourth intron are spliced out, and a ‘wild type’ length transcript (1023 bp) in which the third intron has been spliced out along with one extra base pair. This ‘wild type’ length transcript occurs because the point mutation creates a second ‘AG’ splice acceptor site one base pair downstream of the original site; splicing of the transcript at this site yields a frame shift mutation. To avoid confusion when using PCR amplification to screen for construct expression, we designed new primers (AN334F/AN215R, see Supplementary Table S1 for sequences) for screening transformants that only amplify WT *AP1* transcript, utilizing the single nucleotide difference between WT and the ‘wild type’ length *ap1-1* mutant transcripts.

### The Basis of Functional Divergence between AP1 and FUL

*AP1* and *FUL* have different functions, are expressed in mutually exclusive domains and are divergent in sequence. To determine if the functional divergence between *AP1* and *FUL* is due to changes in regulation, we created promoter swap constructs in which the *FUL* coding sequence is driven by the *AP1* promoter (*pAP1:FUL*) and vice versa (*pFUL:AP1*). If differences in expression underlie the observed functional differences, we would expect our promoter swap constructs to completely complement the corresponding mutant phenotype. If differences in sequence contribute to the functional divergence, we would not expect these constructs to rescue the mutants. We therefore introduced the *pAP1:FUL* construct into the strong *ap1-1* mutant and the *pFUL:AP1* construct into the strong *ful-1* mutant. Plants transformed with the promoter swap constructs were compared to WT and the corresponding mutant as well as positive (*pAP1:AP1 ap1* or *pFUL:FUL ful* ) and negative (empty vector) controls.

#### *FUL* Can Partially Rescue the *ap1* Mutant When Expressed in the *AP1* Domain

**FIGURE 2:**
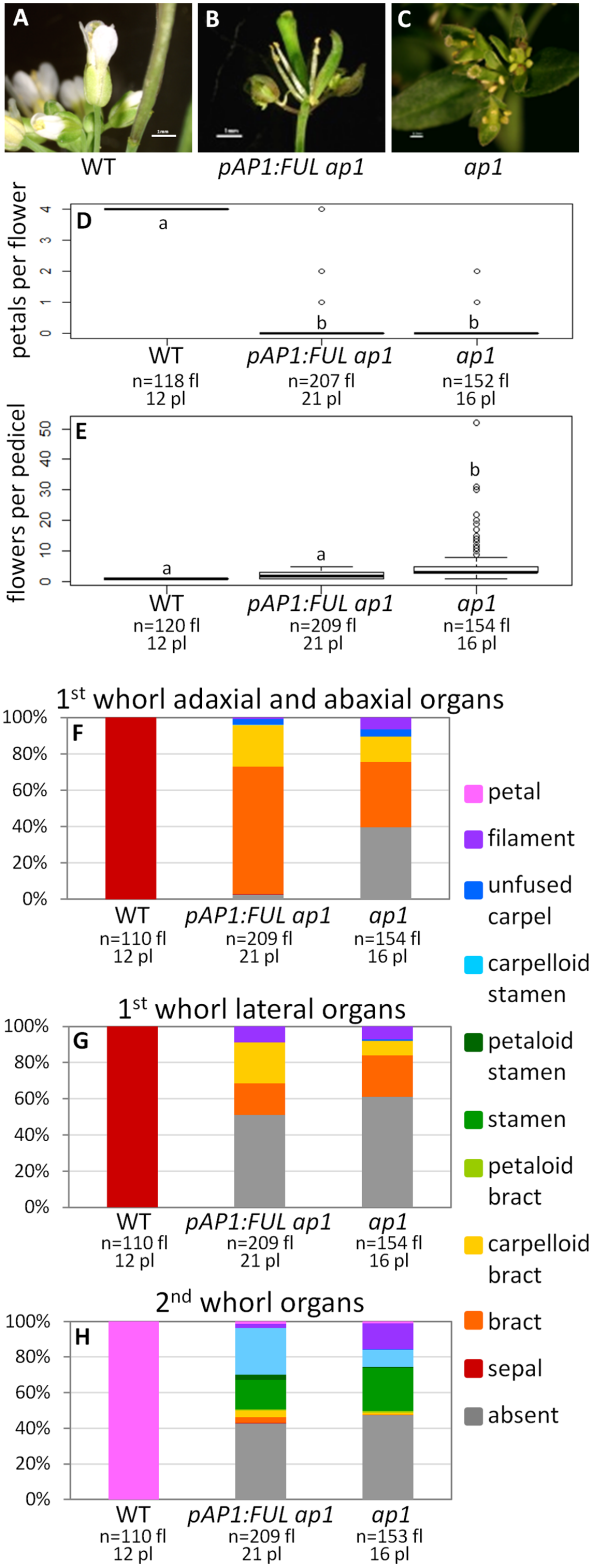
***FUL* can partially substitute for *AP1***. Flowers from wild type (WT) **(A)**, *pAP1:FUL ap1* **(B)**, and *ap1* mutants **(C)**. Box plots showing the number of petals per flower **(D)** and number of flowers per pedicel **(E)** in WT, *pAP1:FUL ap1* lines, and *ap1* mutants. Lowercase letters in box plots denote significance; boxes with the same letter are not significantly different from each other according to ANOVAs and Tukey’s Honest Significant Difference tests and following Bonferroni corrections. Cumulative bar graphs describing the identity of first whorl adaxial and abaxial organs **(F)**, first whorl lateral organs **(G)**, and second whorl organs **(H)** in WT, *pAP1:FUL ap1* lines, and *ap1* mutants. Sample size is noted below each line. The top number is the number of flowers scored, and the bottom number is the number of plants from which these flowers came.

Wild type flowers have one flower per pedicel with four sepals and four petals (**Figures 2A,D–H**). In contrast, *ap1* mutant flowers have multiple flowers per pedicel, and these flowers have predominantly bract-like organs in the first whorl (**Figures 2C,E–G**). However, we observed some carpelloid bracts (in which stigmatic papillae and/or ovules are formed), a few unfused carpels (with stigmatic papillae at the distal end and ovules along the margins), and some filamentous organs in the first whorl in *ap1* mutants (**Figures 2F,G**). Approximately half of both first and second whorl organs are missing (**Figures 2F–H**); when second whorl organs are present, they are mainly stamen-like or filamentous structures, but a few bract-like organs are observed as well (**Figure 2H**). The stamen-like organs are either stamens (with anthers), petaloid stamens (in which petal tissue is fused to the anther), or carpelloid stamens (in which carpelloid structures are either fused to the anther or replace the anther on the filament; **Figure 2H**). Our observations of second whorl organs differ from published descriptions of the *ap1-1* mutant in that we see a greater percentage of organs in this whorl (53% as opposed to 6% in Bowman et al., 1993 and none in Irish and Sussex, 1990); however, the types of organs we observe are consistent with what Bowman et al. (1993) recorded for other *ap1* alleles. The *pAP1:AP1* construct (the positive control) rescues the phenotype to WT; these lines have one flower per pedicel, with petals and predominantly normal sepals, although some sepals have the Y-shaped trichomes characteristic of leaves (Supplementary Figure S3). Empty vector *ap1* lines resemble *ap1* mutants (Supplementary Figure S3).

The promoter swap *pAP1:FUL ap1* lines, in which the *FUL* coding sequence was expressed in the *AP1* domain in an *ap1* mutant background, have mainly bract-like organs in the first whorl; however, we also observed carpelloid bracts, filamentous structures, and a few unfused carpels, similar to *ap1* mutants (**Figures 2B,F,G**). These lines have no petals (ANOVA: *F* = 17530, df = 4, *p* < 2 × 10^-16^), and approximately half of the second whorl organs are missing, but when present, they are mainly stamen-like and filamentous structures, similar to the *ap1* mutant (**Figures 2B,D–H**). However, 98% of adaxial and abaxial first whorl organs are present in the *pAP1:FUL ap1* lines, compared to only 61% in *ap1* mutants (**Figures 2B,F,G**). The identity of the first and second whorl organs of the secondary flowers (which arise from the axils of first whorl organs) is also the same as in *ap1* mutants (ANOVA: *F* = 137.1, df = 2, *p* < 2 × 10^-16^; Supplementary Figure S4). However, in contrast to *ap1* mutants, *pAP1:FUL ap1* lines show a reduced number of flowers per pedicel with an average of 2.04 compared to 5.34 in *ap1* mutants and 1.0 in WT (ANOVA: *F* = 109.2, df = 4, *p* < 2 × 10^-16^; **Figure 2E**). Although *pAP1:FUL ap1* lines are not significantly different from WT in number of flowers per pedicel after Bonferroni correction, they do show some secondary flowers whereas WT plants never do. Although no petals are produced in *pAP1:FUL ap1* lines, the reduced number of flowers per pedicel and the increase in the number of first whorl organs in these lines show that *FUL* can at least partially substitute for *AP1* not only in floral meristem identity, as expected, but also in some elements of floral structure.

#### *AP1* Can Partially Rescue the *ful* Mutant When Expressed in the *FUL* Domain

**FIGURE 3:**
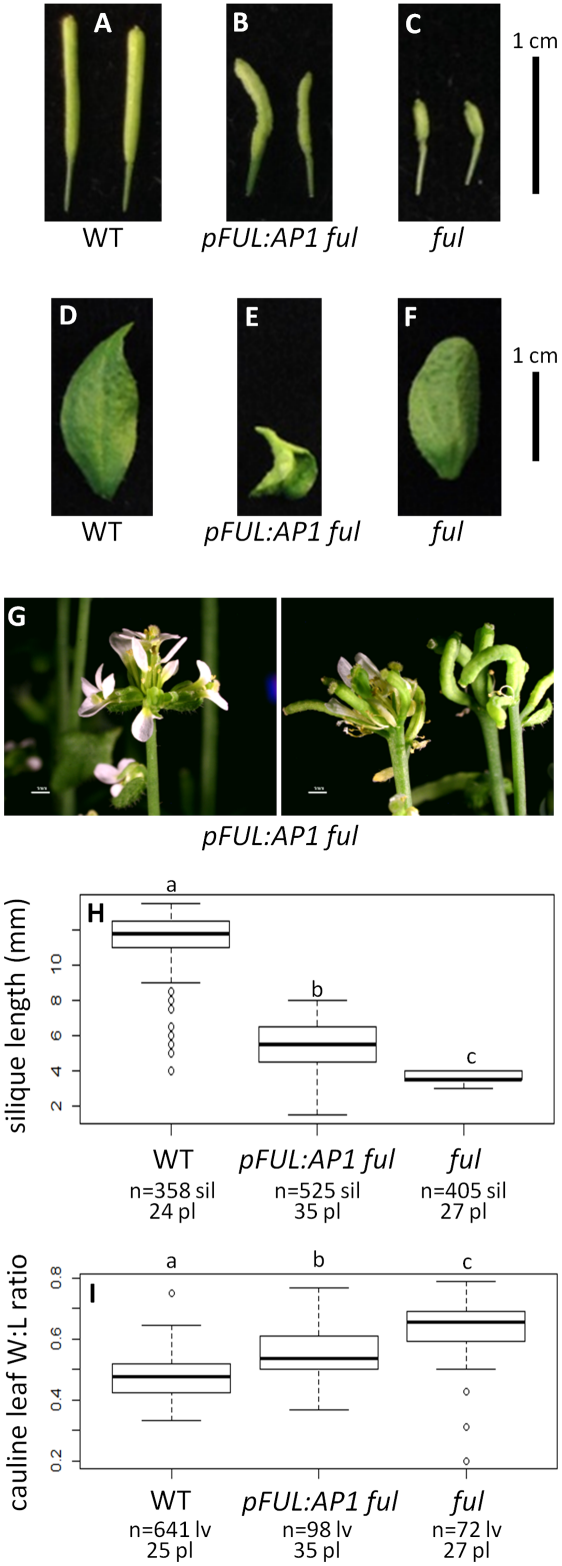
***AP1* can partially substitute for *FUL***. Siliques of WT **(A)**, *pFUL:AP1 ful* **(B)**, and *ful* mutants **(C)**. Cauline leaves of WT **(D)**, *pFUL:AP1 ful* **(E)**, and *ful* mutants **(F)**. Terminal flower phenotype of *pFUL:AP1 ful* lines **(G)**. Box plots showing silique length in millimeters **(H)** and cauline leaf width:length (W:L) ratio **(I)** for WT, *pFUL:AP1 ful* lines, and *ful* mutants. Lowercase letters in box plots denote significance; boxes with the same letter are not significantly different from each other according to ANOVAs and Tukey’s Honest Significant Difference tests and following Bonferroni corrections. Sample size is noted below each line. The top number is the number of siliques or cauline leaves scored, and the bottom number is the number of plants from which they came.

Wild type plants produce siliques with an average length of 11.47 mm and their cauline leaves have a width:length ratio of 0.48 (**Figures 3A,D,H,I**). In contrast, *ful* mutants have siliques with an average length of 3.73 mm and a cauline leaf width:length ratio of 0.63 (**Figures 3C,F,H,I**). The positive control *pFUL:FUL* construct rescues the *ful* mutant phenotype, with an average silique length of 9.63 mm. This is similar to, but slightly shorter than, siliques in WT, but significantly longer than the *ful* mutant siliques (ANOVA: *F* = 3995, df = 4, *p* < 2 × 10^-16^; Supplementary Figure S5). The cauline leaf width:length ratio in *pFUL:FUL ful* lines is 0.45 and is not significantly different from WT (ANOVA: *F* = 106.7, df = 4, *p* < 2 × 10^-16^; Supplementary Figure S5). Empty vector *ful* lines resemble *ful* mutants (Supplementary Figure S5).

The *pFUL:AP1 ful* lines, in which the *AP1* coding sequence is expressed in the *FUL* domain in a *ful* mutant background, have lumpy, sometimes curved siliques that are significantly longer (5.30 mm) than *ful* mutants, but significantly shorter than WT (ANOVA: *F* = 3995, df = 4, *p* < 2 × 10^-16^; **Figures 3B,H**). The *pFUL:AP1 ful* lines have curled cauline leaves with an average width:length ratio of 0.55; this is significantly larger than the WT average of 0.48, but significantly smaller than the average of 0.63 seen in *ful* mutants (ANOVA: *F* = 106.7, df = 4, *p* < 2 × 10^-16^; **Figure 3I**). The *pFUL:AP1 ful* lines have terminal flowers (**Figure 3G**), which are not present in WT plants; these likely result from *AP1* expression in the inflorescence meristem under the control of the *FUL* promoter. Longer siliques and relatively narrower cauline leaves in the *pFUL:AP1 ful* lines show that *AP1* can partially substitute for *FUL* when expressed in the *FUL* domain.

### The Function of Conserved Motifs

#### The Farnesylation Motif is not Required for *AP1* Function

**FIGURE 4:**
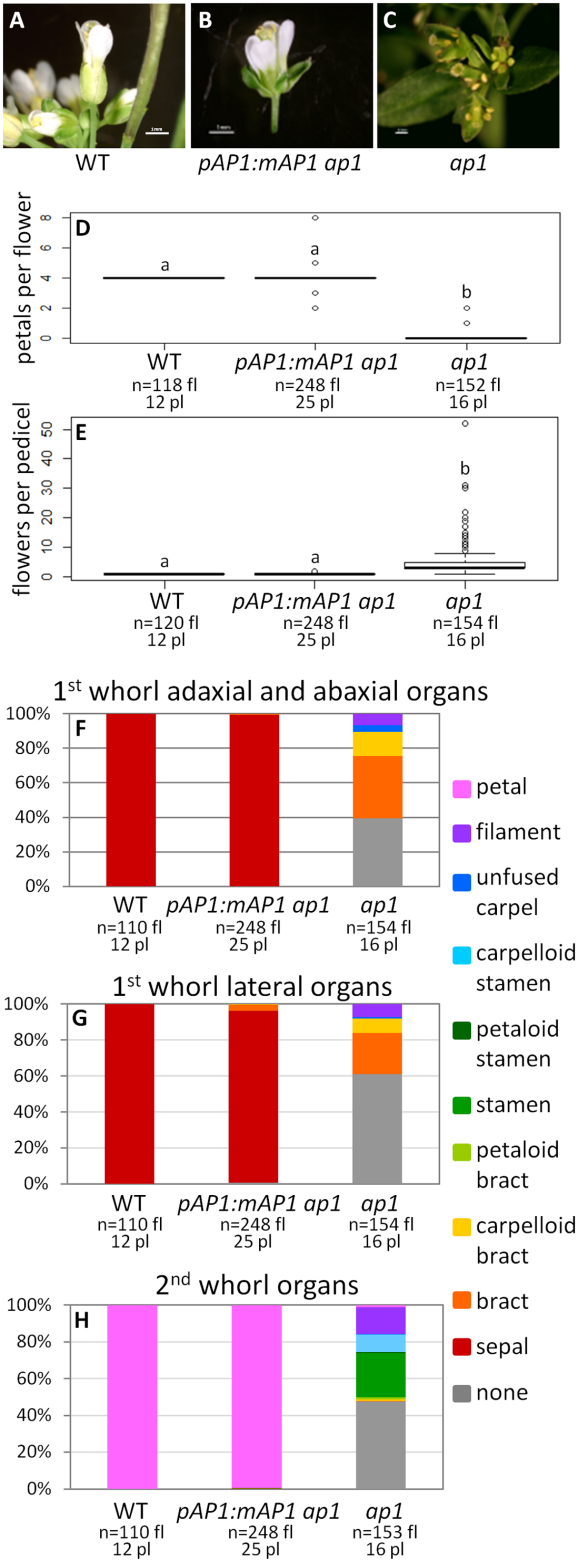
**AP1 farnesylation motif is not necessary for protein function**. Flowers from WT **(A)**, *pAP1:mAP1 ap1* **(B)**, and *ap1* mutants **(C)**. Box plots showing the number of petals per flower **(D)** and the number of flowers per pedicel **(E)** for WT, *pAP1:mAP1 ap1*, and *ap1* mutants. Lowercase letters in box plots denote significance; boxes with the same letter are not significantly different from each other according to ANOVAs and Tukey’s Honest Significant Difference tests and following Bonferroni corrections. Cumulative bar graphs describing the identity of first whorl adaxial and abaxial organs **(F)**, first whorl lateral organs **(G)**, and second whorl organs **(H)** in WT, *pAP1:mAP1 ap1* lines, and *ap1* mutants. Sample size is noted below each line. The top number is the number of flowers scored, and the bottom number is the number of plants from which these flowers came.

To determine if the AP1 farnesylation motif is required for protein function, we generated a construct in which the receptor cysteine was mutated to a serine (*pAP1:mAP1*; **Figure 1**), which prevents the attachment of the farnesyl molecule. The *pAP1:mAP1 ap1* lines resemble WT; they have only one flower per pedicel (ANOVA: *F* = 138.5, df = 4, *p* < 2 × 10^-16^; **Figures 4A,B,D–H**), and those flowers have petals (ANOVA: *F* = 18495, df = 4, *p* < 2 × 10^-16^) and sepals (sometimes with Y-shaped trichomes). The fact that the mutated AP1 protein can complement the *ap1* mutant shows that farnesylation of the AP1 protein is not necessary for proper protein function.

#### FUL Protein Function is Largely not Dependent on the FUL-like Motif

We examined whether the FUL-like motif is necessary for FUL protein function by creating three mutated coding sequences: *tFUL*, which creates a protein that is truncated just before the FUL-like motif; *mFULp*, in which the proline in the FUL-like motif is replaced with a serine; and *mFULw*, in which the tryptophan is replaced with a glutamine. The tryptophan in the fourth position of the motif is strictly conserved in all proteins from the AP1/FUL, SEP, and AGL6 lineages, and the proline in the second position is conserved across angiosperm sequences (Litt and Irish, 2003). In both mFULp and mFULw, a non-polar amino acid was replaced with a polar amino acid, disrupting the hydrophobic motif.

**FIGURE 5:**
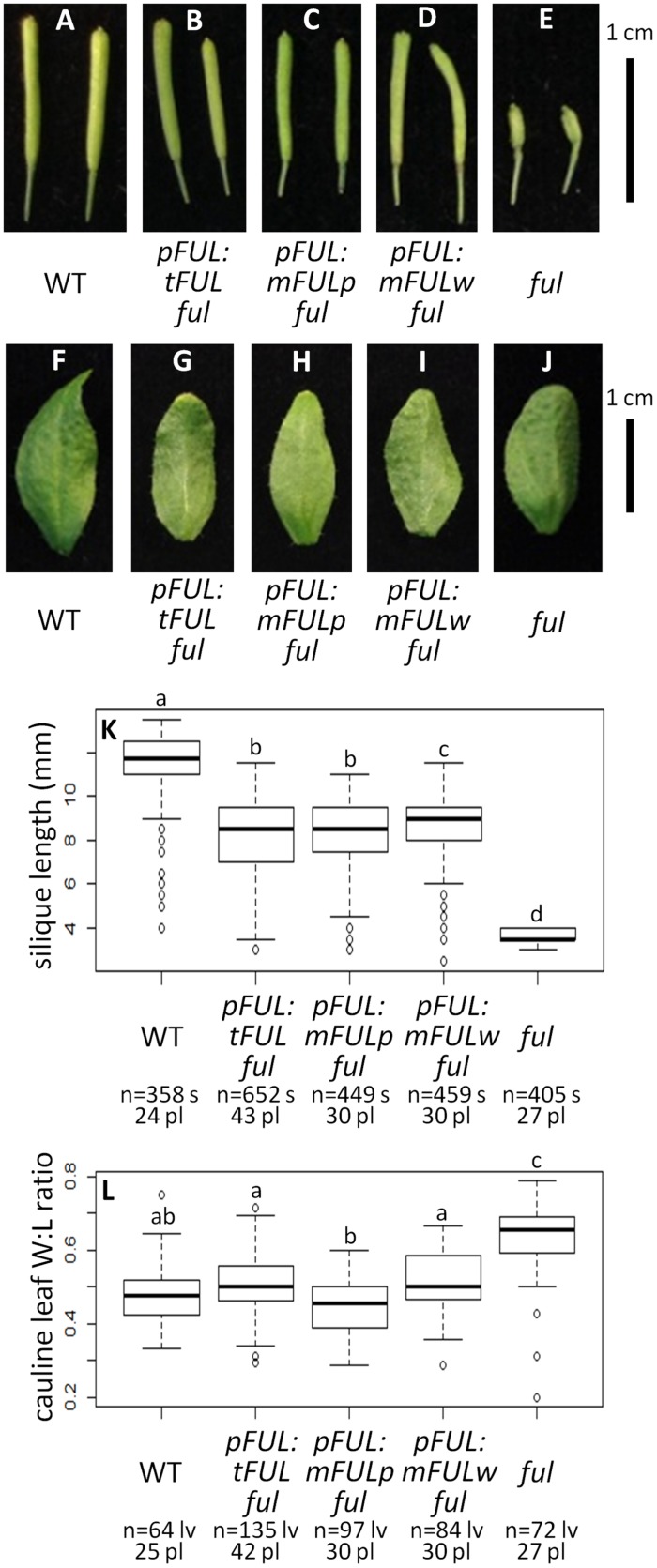
***FUL*-like motif plays a minor role in silique elongation**. Siliques from WT **(A)**, *pFUL:tFUL ful* **(B)**, *pFUL:mFULp ful* **(C)**, *pFUL:mFULw ful* **(D)**, and *ful* mutants **(E)**. Photographs of cauline leaves from WT **(F)**, *pFUL:tFUL ful* **(G)**, *pFUL:mFULp ful* **(H)**, *pFUL:mFULw ful* **(I)**, and *ful* mutants **(J)**. Box plots showing silique length in millimeters **(K)** and cauline leaf width:length (W:L) ratio **(L)** for WT, *pFUL:tFUL ful*, *pFUL:mFULp ful*, *pFUL:mFULw ful*, and *ful* mutants. Lowercase letters in box plots denote significance; boxes with the same letter are not significantly different from each other according to ANOVAs and Tukey’s Honest Significant Difference tests and following Bonferroni corrections. Sample size is noted below each line. The top number is the number of siliques or cauline leaves scored, and the bottom number is the number of plants from which they came.

Average silique length was similar in all three sets of plants transformed with mutated *FUL* constructs: *pFUL:tFUL ful* (8.10 mm), *pFUL:mFULp ful* (8.09 mm), and *pFUL:mFULw ful* (8.52 mm). These lengths were significantly longer than *ful* mutants (3.73 mm), but significantly shorter than WT (11.47 mm; ANOVA: *F* = 1895, df = 6, *p* < 2 × 10^-16^; **Figures 5A–E,K**), suggesting that this conserved amino acid motif may have a minor role in silique elongation. The cauline leaf width:length ratio in all three mutated *FUL ful* lines was not significantly different than WT (0.48); however, the ratio in *pFUL:mFULp ful* lines (0.45) is significantly smaller than in *pFUL:tFUL ful* (0.51) and *pFUL:mFULw ful* (0.57) lines (ANOVA: *F* = 108.8, df = 6, *p* < 2 × 10^-16^; **Figures 5F–J,L**). This observation, that a protein lacking the proline is more successful at complementing the phenotype than one lacking the tryptophan or lacking the motif entirely, suggests that the proline plays a less significant role than the tryptophan in protein function. Nonetheless, none of the ratios were significantly different from WT, suggesting that this motif is not required for proper cauline leaf development. These results, which show that the mutated protein constructs complement the *ful* cauline leaf phenotype and partially complement the silique defect, suggest that the FUL-like motif plays only a minor role in FUL protein function.

### Protein–protein Interactions

MADS-domain proteins are thought to act in multimeric complexes (Egea-Cortines et al., 1999; Honma and Goto, 2001; Theissen, 2001; Theissen and Saedler, 2001; Melzer and Theissen, 2009; Melzer et al., 2009). Yalovsky et al. (2000) suggested that farnesylation, which adds a hydrophobic tail to the C terminus of the AP1 protein, might mediate such interactions. Furthermore, the conserved FUL-like motif consists of hydrophobic amino acids, suggesting a possible role in protein–protein interactions. We therefore conducted yeast two-hybrid experiments to determine whether mutations in these motifs disrupt known protein–protein interactions. AP1 is capable of autoactivation, so yeast two-hybrid constructs were made in which the proline- and glutamine-rich regions and activation domain were removed from the C terminus of the protein for both AP1 and mAP1 (Supplementary Figure S2).

**FIGURE 6:**
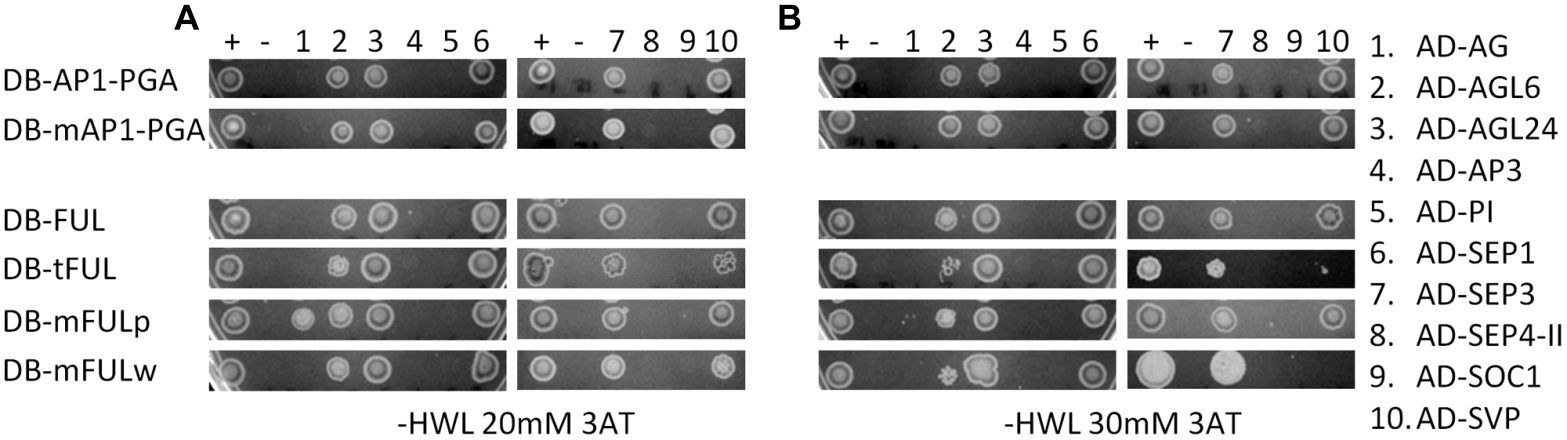
**The FUL-like, but not the farnesylation, motif may play a role in mediating protein interactions**. Interactions in yeast on -HWL 20 mM 3AT **(A)** and -HWL 30 mM 3AT **(B)** plates after 6 days of growth. Mutated proteins were compared with WT AP1 and FUL proteins to determine whether altering or abolishing the conserved motifs disrupted protein–protein interactions. Numbers above the images refer to proteins listed in the key on the right hand side. Constructs fused to the binding domain are labeled “DB”; constructs fused to the activation domain are labeled “AD.” Proteins and controls are described in the text. + and – indicate positive and negative (empty vector) control. AP1-PGA, mAP1-PGA = AP1 protein and AP1 protein with mutated farnesylation motif lacking the proline-rich (P), glutamine-rich (G), and activation (A) domains to abolish autoactivation (see Supplementary Figure S2A for diagram).

In our experiments, AP1 and FUL proteins interacted with AGL6, AGL24, SEP1, SEP3, and SVP, but not AG, AP3, PI, SEP4-II, or SOC1 (**Figure 6**). The mAP1 protein interactions were identical (**Figure 6**), indicating that, at least in yeast, the farnesylation motif is not required for dimerization with the MADS-domain proteins we investigated. FUL proteins with mutated motifs also have largely the same interaction partners as WT when grown on lower stringency plates (-HLW with 20 mM 3AT). An exception is that mFULp, in which the proline of the FUL-like motif is replaced by a serine, interacts with AG (**Figure 6A**), whereas WT protein does not, under our conditions. However, this interaction is no longer seen on higher stringency plates (-HLW with 30 mM 3AT; **Figure 6B**). The interactions of tFUL (truncated) and mFULw (tryptophan replaced with glutamine) with AGL6 and SVP are also weaker or absent on the higher stringency plates (**Figure 6B**), suggesting that the FUL-like motif may play at least some role in protein–protein interactions.

## Discussion

### Both Changes in Regulation and Coding Sequence Underlie Functional Differentiation of *AP1* and *FUL*

Our results show that the *FUL* coding sequence can partially complement the *ap1* mutant when expressed in the *AP1* domain and that the *AP1* coding sequence can partially rescue the *ful* mutant when expressed in the *FUL* domain (**Figures 2** and **3**). These results suggest that the divergence in function between these two genes is the result of changes in coding sequence as well as changes in regulation. Our *pFUL:AP1 ful* lines show partial rescue of the silique length and cauline leaf shape defects of the *ful* mutant (**Figures 3A–F,H,I**), showing that the AP1 protein has some ability, albeit limited, to substitute for FUL. Our *pAP1:FUL ap1* lines had fewer flowers per pedicel, showing that *FUL* can moderate the inflorescence meristem defects of *ap1* (**Figure 2E**). This is consistent with the fact that *FUL*, as well as *AP1*, is capable of promoting floral meristem identity (Ferrandiz et al., 2000). The *pAP1:FUL ap1* lines also had an increase in first whorl organ number. However, no petals are formed in these lines (**Figure 2D**), showing that FUL does not have all of the functional capabilities of AP1. Previous experiments expressing a *FUL*-like gene from the grass *Lolium temulentum* in the *Arabidopsis ap1* mutant under the control of the *AP1* promoter similarly showed that this gene could not rescue the petal defect but was able to partially complement the defects in flower number per pedicel and first whorl organ number (Gocal et al., 2001). The fact that expression of either *AP1* or *FUL* in the domain of the other produces only partial mutant complementation indicates that the proteins are not functionally equivalent, and that differences in sequence do have functional consequences.

### Conserved AP1 and FUL Amino Acid Motifs are not Necessary for Protein Function

#### AP1 farnesylation motif

Our mutated *AP1 ap1* lines, in which the receptor cysteine of the farnesylation motif was replaced with serine thus preventing farnesylation of the AP1 protein, show complete complementation of the mutant phenotype (**Figure 4**). The only exception is that sepals sometimes have Y-shaped trichomes, which is also seen in our *pAP1:AP1 ap1* positive control lines. These results suggest that this post-translational modification of the AP1 protein, which has been demonstrated to occur *in planta* (Yalovsky et al., 2000), is not necessary for proper AP1 function. Yalovsky et al. (2000) generated the same mutated AP1 protein, in which the receptor cysteine was replaced with serine, and expressed it in WT *Arabidopsis* plants under the cauliflower mosaic virus (CaMV) 35S promoter (Benfey et al., 1989). Although their mutated AP1 lines flowered early, similar to what is seen when WT AP1 is overexpressed, these lines failed to show the terminal flower phenotype that is typical of AP1 overexpression (Mandel and Yanofsky, 1995b). Instead they displayed novel phenotypes, including increased density of trichomes on rosette leaves and sepals and sometimes lack of chlorophyll in leaves and inflorescences; this led them to conclude that farnesylation played an important role in AP1 function (Yalovsky et al., 2000). Because overexpression phenotypes may not reflect the function of a protein during normal plant development, we instead expressed the mutated *AP1* sequence under the control of the WT *AP1* promoter in the *ap1* mutant to see if it could replace the WT protein. Our results suggest that it can, and that addition of a farnesyl molecule is not required for normal AP1 function. Nevertheless, Yalovsky et al. (2000) did show that the protein is farnesylated in *Arabidopsis*, thus the purpose of this modification remains unknown.

Evidence from studies with *AP1/FUL* genes from other species also suggests that this post-translational modification is not required for AP1 function. The AP1 ortholog in pea, PEAM4, lacks a farnesylation motif, but can restore petal production when constitutively expressed in *Arabidopsis ap1-1* mutants (Berbel et al., 2001). The average number of petals per flower in these lines is less than that seen in WT or 35S:*AP1* lines (Berbel et al., 2001), but this may be due to additional sequence changes that arose since the divergence of *Arabidopsis* and pea. Similarly, overexpression of a *euFUL* gene from tobacco and *FUL*-like genes from *Lilium* and rice, which all lack the farnesylation motif, produced petals; although, in fewer numbers than in WT plants (Jang et al., 2002; Chen et al., 2008).

*Arabidopsis* has another *AP1* paralog, *CAULIFLOWER* (*CAL*), which arose from a more recent duplication than *AP1* and *FUL*. *CAL* possesses a farnesylation motif, but kinetic analyses suggest that it is unlikely to be farnesylated *in planta* (Yalovsky et al., 2000). When expressed under the *AP1* promoter, *CAL* cannot rescue the *ap1-1* mutant phenotype; however, chimeric proteins, in which the M-, I-, and K-domains of AP1 are fused to the C-domain of CAL, nearly completely rescue *ap1-1* (Alvarez-Buylla et al., 2006). This suggests that the inability of CAL to substitute for AP1 is due to sequence in the M-, I-, and K-domains, and not the C-terminal domain that contains the farnesylation motif.

#### FUL-like Motif

The *ful* mutant phenotype is nearly completely rescued by all three of our mutated *FUL* constructs (**Figure 5**), in which either the entire motif was absent or the most highly conserved residues were substituted. This suggests that the FUL protein can function nearly normally without the FUL-like motif; however, silique length in all three mutated *FUL ful* lines is significantly shorter than WT (**Figures 5A–E,K**), indicating that the FUL-like motif plays at least some role in silique elongation.

The FUL-like motif is highly conserved not only in all euFUL proteins, but also in the FUL-like proteins found in plant lineages outside the core eudicots (which predate the euAP1/euFUL duplication), and in the closely related *SEP* and *AGL6* gene clades. These latter genes are also implicated in flowering and floral development, and arose via duplication from the same lineage as *AP1/FUL* (Litt and Irish, 2003). Only the *AGL6* lineage predates angiosperms, being found in gymnosperms as well; *AP1/FUL* and *SEP* genes are restricted to flowering plants and are required for flowering (Theissen et al., 2000; Litt and Irish, 2003). The tryptophan in the fourth position of the six amino acid motif appears to be strictly conserved across not only FUL and FUL-like proteins, but also the SEP and AGL6 lineages (Litt and Irish, 2003). This suggests an important function for this residue; however, our results suggest only a minor role in silique elongation. The proline in the second position is conserved in angiosperm euFUL and FUL-like proteins (Litt and Irish, 2003), and similarly, only seems to play a minor role in silique elongation. Our results suggest that the mutated proline transcript may be better able to complement the cauline leaf width:length ratio defect of the mutant; nonetheless, none of these lines were significantly different from WT for this trait (**Figures 5F–J,L**). The fact that SEP proteins have this same motif, and that several SEP proteins are co-expressed with FUL (de Folter et al., 2005), suggests that SEP proteins may be able to substitute to some extent, although certainly not completely, for FUL, thereby masking the significance of the loss or alteration of the FUL-like motif in FUL.

Our promoter swap experiments show that sequence differences between AP1 and FUL have functional consequences; however, our results suggest that the relevant differences are not those of the highly conserved amino acid motifs present in the C-terminal domain. It goes against accepted wisdom to suggest that highly conserved motifs do not have functional significance. However, studies with a chimeric protein, in which the MADS and I domains of AP1 were fused to the K and C domains of AGAMOUS (AG), show that in fact that the specific sequence of the entire C terminus may not be required. This chimeric protein can provide nearly complete complementation of the *ap1-1* mutant phenotype when driven by the *AP1* promoter (Krizek et al., 1999), yet AG has entirely different conserved amino acid motifs in its C-terminal domain (Kramer et al., 2004). Studies with truncated APETALA3 (AP3) and PISTILLATA (PI) proteins that lack the conserved C-terminal motifs characteristic of those lineages (Piwarzyk et al., 2007) confirm that the conspicuous motifs of MADS-domain proteins are less significant than assumed. This raises the question of why they are so highly conserved, particularly as the rest of the C-terminal domain tends to be highly variable, even among closely related species.

#### Altering or Abolishing Conserved Motifs Affects some Protein Interactions in Yeast

Our yeast-two hybrid experiments reveal that abolishing the AP1 farnesylation motif does not alter protein–protein interactions (**Figure 6**). This indicates that the farnesylation motif is not necessary for dimerization with the MADS-domain proteins we tested; however, it is still possible that this motif is involved in interaction with other proteins or plays a role in the formation of multimeric complexes. In addition, it has not been shown that AP1 is farnesylated in yeast; although yeast has the machinery to perform this post-translational modification (Omer and Gibbs, 1994). Nevertheless, our transgenic experiments, which indicate that this motif is not required for protein function, support the idea that it is not required to mediate functionally relevant protein interactions.

In contrast, tFUL (in which the FUL protein terminates before the FUL-like motif) and mFULw (in which tryptophan is replaced with glutamine) have weaker interactions with AGL6 and SVP than are seen with FUL (**Figure 6B**). This indicates that the FUL-like motif may play a role in some protein–protein interactions, consistent with its hydrophobic nature. FUL-SVP heterodimers have been implicated in the regulation of meristem identity in the vegetative-reproductive transition (Balanzà et al., 2014). The specific role of FUL-AGL6 interactions has not been documented; however, both genes are co-expressed in floral development and are implicated in the regulation of flowering (Ohmori et al., 2009; Rijpkema et al., 2009; Thompson et al., 2009; Koo et al., 2010; Yoo et al., 2011). These interactions may be at least partly dependent on the FUL-like motif. We did not observe defects in flowering time in our transgenic experiments with mutated FUL proteins, suggesting that weak interactions may be sufficient to produce the appropriate developmental outcomes; alternatively, the program that promotes flowering contains significant redundancy which may compensate for any loss of interaction.

Our yeast-two hybrid results differ from those previously published: in contrast to our findings, de Folter et al. (2005) found that FUL did not interact with SVP, but did interact with AG, and that both FUL and AP1 interacted with SEP4-II and SOC1. However, van Dijk et al. (2010) observed FUL-SVP interaction, similar to what we found, and Balanzà et al. (2014) confirmed this interaction *in planta*. Although this type of study allows comparison with previous published protein interaction studies, yeast-two hybrid experiments are known to produce both false positives and negatives, and to be sensitive to experimental conditions (Legrain and Selig, 2000); therefore, it is not surprising that results may vary. Also, the relevance of observed interactions to *in planta* processes must be verified.

Our yeast-two hybrid experiments were limited to MADS-domain proteins, but AP1 and FUL interact with proteins of other families as well, and it is possible that these interactions may be influenced by the C-terminal motifs. Nevertheless, the data from plants transformed with our mutated protein constructs suggest that any effects that are present do not produce significant phenotypic defects. This may be due to redundancy, particularly in the case of FUL, or it may simply be that these motifs are not as critical for proper function as assumed.

### Duplicate Genes Promote both Diversity and Redundancy in Developmental Networks

Functional divergence of gene duplicates can result in maintenance of both copies in the genome and lead to new gene functions that can produce novel phenotypes and increased organismal complexity (Ohno, 1970; Freeling and Thomas, 2006). Functional divergence can be based on changes in expression, changes in sequence, or both. Our experiments indicate that in the case of the *Arabidopsis* paralogs *AP1* and *FUL*, the third option has occurred; changes in both regulation and coding sequence have driven functional divergence. More recent paralogs, *AP1* and *CAL*, share a similar expression pattern, and *CAL* is completely redundant with *AP1* function; although, the reverse is not true. Loss of *CAL* function enhances the loss of floral meristem identity (Kempin et al., 1995), but *CAL* is not able to complement the floral organ defects of the *ap1* mutant when driven by the *AP1* promoter (Alvarez-Buylla et al., 2006). In fact, *CAL* may be gradually losing its function; it has a higher rate of non-synonymous substitutions than *AP1* (Liljegren et al., 1999) and fewer MADS-domain protein interaction partners than *AP1* (de Folter et al., 2005). Amino acid differences at two positions between *AP1* and *CAL* have been shown to account for a large portion of the interaction differences observed between the two proteins, and swapping these residues yielded gain or loss of some protein interaction partners in a yeast system (van Dijk et al., 2010). *FUL* shares one of these amino acid residues with *AP1*, which may explain why *FUL* is able to partially rescue the *ap1* mutant when expressed in the *AP1* domain (**Figure 2**), whereas *CAL* cannot (Alvarez-Buylla et al., 2006).

Intriguingly, all three of these genes that are related by duplication retain the function of promoting floral meristem identity, although to different extents and in different contexts. In WT development, *FUL* does not play this role because it is excluded from the floral meristem by *AP1* (Mandel and Yanofsky, 1995a), providing an example of how changes in regulation can result in functional divergence between duplicates. *AP1* and *CAL* are expressed in similar domains, but *AP1* has functional capabilities that *CAL* does not have (Kempin et al., 1995; Ferrandiz et al., 2000); thus sequence differences must be the differentiating factor. Nevertheless, all three genes are capable of contributing to floral meristem identity in a redundant fashion. This evidence supports the hypothesis that duplicate genes not only diversify and create novel complexity within developmental systems, but also that they strengthen already existing pathways to ensure robustness in important developmental processes, such as the transition to flowering.

## Author Contributions

AL developed the project. EM performed most of the experiments. AM cloned the AP1 promoter and FUL coding sequence, created the yeast-two hybrid constructs, and performed yeast-two hybrid experiments. EM and AL wrote the manuscript.

## Conflict of Interest Statement

The authors declare that the research was conducted in the absence of any commercial or financial relationships that could be construed as a potential conflict of interest.
