# Supplementary material for: Functional Divergence of APETALA1 and FRUITFULL is due to Changes in both Regulation and Coding Sequence
Source: Front Plant Sci. 2015 Dec 2;6:1076. doi: 10.3389/fpls.2015.01076 (PMC4667048; doi:10.3389/fpls.2015.01076)
Supplement: Supplementary file 1 [file Supplementary_Materials_and_Methods.DOCX]

**Supplementary Material and Methods**

**DNA extraction**

We performed DNA extraction as follows. Tissue was collected in liquid nitrogen and ground using a micropestle. 700 μL of DNA extraction buffer (0.2M Tris HCl, 0.25M NaCl, 0.025M EDTA, 0.5% SDS) was added, tubes were vortexed and incubated at room temperature for 10 minutes. Tubes were centrifuged at room temperature for 10 min. The supernatant was transferred to a new tube. One tenth volume of 3M sodium acetate, pH 5.2 and 500 μL of isopropanol were added to each tube and inverted to mix. Tubes were centrifuged at room temperature for 10 minutes, and the supernatant was discarded. The pellet was washed with 500 μL of 70% ethanol. Tubes were centrifuged at room temperature for five minutes, and the supernatant was discarded. The pellet was air dried and then resuspended in 20 μL TE buffer.

**PCR conditions**

The PCR conditions for creation of all constructs and for screening PCRs are as follows.

**Cloning of promoters and coding sequences**

***AP1* promoter + 5’UTR:** 94°C for 2 minutes followed by 30 cycles of 94°C for 30 seconds, 60°C for 30 seconds, and 72°C for 1 minute, and a final extension of 72°C for 10 minutes.

***FUL* promoter + 5’ UTR:** 94°C for 3 minutes followed by 30 cycles of 94°C for 1 minutes, 48°C for 30 seconds, and 68°C for 4 minutes, with a final extension of 68°C for 20 minutes

***AP1* coding sequence + 3’ UTR:**  94°C for 3 minutes followed by 35 cycles of 94°C for 1 minute, 50°C for 1 minute, and 72°C for 1 minute, and a final extension of 72°C for 7 minutes

***FUL* coding sequence + 3’ UTR:** 92°C for 2 minutes followed by 30 cycles of 92°C for 30 seconds, 59°C for 40 seconds, and 72°C for 30 seconds, and a final extension of 72°C for 6 minutes

**Mutated coding sequences through site-directed mutagenesis and chimeric PCR**

***mAP1* coding sequence + 3’ UTR:**

**PCR1:** 94°C for 3 minutes followed by 30 cycles of 94°C for 1 minute, 50°C for 1 minute, and 72°C for 1 min, and a final extension of 72°C for 7 minutes

**PCR2:** 94°C for 3 minutes followed by 30 cycles of 94°C for 1 minute, 54°C for 1 minute, and 72 °C for 30 seconds, and a final extension of 72°C for 7 minutes

**PCR3:** 94°C for 3 minutes followed by 30 cycles of 94°C for 1 minute, 50°C for 1 minute, and 72°C for 90 seconds, and a final extension of 72°C for 7 minutes

***tFUL* coding sequence + 3’ UTR:**

**PCRs 1+2:** 92°C for 2 minutes followed by 35 cycles of 92°C for 30 seconds, 50°C for 30 seconds, and 72°C for 40 seconds, and a final extension of 72°C for 10 minutes

**PCR3:** 92°C for 2 minutes followed by 35 cycles of 92°C for 30 seconds, 59°C for 40 seconds, and 72°C for 40 seconds, and a final extension of 72°C for 10 minutes

***mFULp* and *mFULw* coding sequences + 3’ UTR:**

**PCR1:** 95°C for 5 minutes followed by 35 cycles of 95°C for 30 seconds, 69°C for 30 seconds, and 72°C for 3 minutes, and a final extension of 72°C for 10 minutes

**PCR2:** 95°C for 5 minutes followed by 35 cycles of 95°C for 30 seconds, 58°C for 30 seconds, and 72°C for 3 minutes, and a final extension of 72°C for 10 minutes

**PCR3:** 92°C for 2 minutes followed by 30 cycles of 92°C for 15 seconds, 59°C for 30 seconds, and 72°C for 3 minutes, and a final extension of 72°C for 7 minutes

**Chimeric PCRs to link promoters and coding sequences**

***pAP1:AP1* and *pAP1:mAP1*:**

**PCR1:** 94°C for 3 minutes followed by 30 cycles of 94°C for 1 minute, 40°C for 1 minute and 72°C for 2 minutes, and a final extension of 72°C for 7 minutes

**PCR2:** 94°C for 3 minutes followed by 30 cycles of 94°C for 1 minute, 52°C for 1 minute, and 72°C for 1 minute, and a final extension of 72°C for 7 minutes

**PCR3:** 94°C for 3 minutes followed by 30 cycles of 94°C for 1 minute, 47°C for 1 minute, and 72°C for 3 minutes, and a final extension of 72°C for 7 minutes

***pAP1:FUL*:**

**PCR1:** 94°C for 3 minutes followed by 30 cycles of 94°C for 1 minute, 40°C for 30 seconds, and 72°C for 2 minutes, and a final extension of 72°C for 7 minutes

**PCR2:** 94°C for 3 minutes followed by 30 cycles of 94°C for 1 minute, 55°C for 30 seconds, and 72°C for 1 minute, and a final extension of 72°C for 7 minutes

**PCR3:** 94°C for 3 minutes followed by 30 cycles of 94°C for 1 minute, 55°C for 1 minute, and 72°C for 3 minutes, and a final extension of 72°C for 7 minutes

***pFUL:FUL*, *pFUL:AP1*, *pFUL:tFUL*, *pFUL:mFULp*, and *pFUL:mFULw*:**

**PCR1:** 94°C for 3 minutes followed by 30 cycles of 94°C for 1 minute, 40°C for 1 minute, and 72°C for 30 seconds, and a final extension of 72°C for 7 minutes

**PCR2:** 94°C for 3 minutes followed by 30 cycles of 94°C for 1 minute, 48°C for 1 minute, and 72°C for 1 minute, and a final extension of 72°C for 7 minutes

**PCR3:** 94°C for 3 minutes followed by 30 cycles of 94°C for 1 minute, 50°C for 1 minute, and 72°C for 90 seconds, and a final extension of 72°C for 7 minutes

**Transformant screening PCRs**

**Kanamycin:** 94°C for 3 minutes followed by 40 cycles of 94°C for 30 seconds, 57°C for 30 seconds, and 72°C for 30 seconds, and a final extension of 72°C for 7 minutes

**Hygromycin:** 94°C for 3 minutes followed by 35 cycles of 94°C for 30 seconds, 52°C for 30 seconds, and 72°C for 30 seconds, and a final extension of 72°C for 7 minutes

**Actin:** 94°C for 3 minutes followed by 37 cycles of 94°C 30 seconds, 57°C for 30 seconds, and 72°C for 30 seconds, and a final extension of 72°C for 7 minutes

***pAP1:AP1*, *pAP1:FUL*, and *pAP1:mAP1* screen:** 94°C for 3 minutes followed by 40 cycles 94°C for 30 seconds, 57°C for 30 seconds, and 72°C for 30 seconds, and a final extension of 72°C for 7 minutes

***pFUL:FUL*, *pFUL:AP1*, *pFUL:tFUL*, *pFUL:mFULp*, and *pFUL:mFULw* screen:** 94°C for 3 minutes followed by 35 cycles of 94°C for 30 seconds, 58°C for 30 seconds, and 72°C for 1 minute, and a final extension of 72°C for 7 minutes

***pFUL:AP1*:** 94°C for 3 minutes followed by 35 cycles 94°C for 30 seconds, 55°C for 30 seconds, and 72°C for 30 sec, and a final extension of 72°C for 7 minutes

***pFUL:tFUL*:** 94°C for 3 minutes followed by 35 cycles of 94°C for 30 seconds, 55°C for 30 seconds, and 72°C for 30 seconds and a final extension of 72°C for 7 minutes

***pFUL:mFULp*:** 94°C for 3 minutes followed by 35 cycles of 94°C for 30 seconds, 67°C for 30 seconds, and 72°C for 30 seconds, and a final extension of 72°C for 7 minutes

***pFUL:mFULw*:** 94°C for 3 minutes followed by 35 cycles of 94°C for 30 seconds, 57°C for 30 seconds, and 72°C for 30 seconds, and a final extension of 72°C for 7 minutes

**Yeast-two hybrid constructs**

**AG:** 95°C for 2 minutes followed by 30 cycles of 95°C for 30 seconds, 64°C for 30 seconds, and 72°C for 1 minute, and a final extension of 72°C for 7 minutes

**AGL6:** 98°C for 1 minute followed by 30 cycles of 98°C for 30 seconds, 64°C for 30 seconds, and 72°C for 1 minute, and a final extension of 72°C for 7 minutes

**SEP3:** 98°C for 2 minutes followed by 30 cycles of 98°C for 20 seconds, 56°C for 30 seconds, and 72°C for 30 seconds, and a final extension of 72°C for 7 minutes

**SEP4-II:** 98°C for 1 minute followed by 30 cycles of 98°C for 20 seconds, 58°C for 20 seconds, and 72°C for 30 seconds, and a final extension of 72°C for 5 minutes

**SVP:** 95°C for 2 minutes followed by 30 cycles of 95°C for 30 seconds, 64°C for 30 seconds, 72°C for 1 minute, and a final extension of 72°C for 7 minutes

**mutated FUL sequences:** 95°C for 2 minutes followed by 30 cycles of 95°C for 30 seconds, 57°C for 30 seconds, and 72°C for 1 minute, and a final extension of 72°C for 7 minutes

**chimeric PCRs for AP1-PGA and mAP1-PGA:**

**PCRs 1+2+3:** 98°C for 1 minute followed by 33 cycles of 98°C for 20 seconds, 59°C for 20 seconds, and 72°C for 30 seconds, and a final extension of 72°C for 7 minutes
