# Supplementary material for: Functional Divergence of APETALA1 and FRUITFULL is due to Changes in both Regulation and Coding Sequence
Source: Front Plant Sci. 2015 Dec 2;6:1076. doi: 10.3389/fpls.2015.01076 (PMC4667048; doi:10.3389/fpls.2015.01076)
Supplement: Supplementary file 2 [file Table_1.DOCX]

**Supplementary Table 1: Primer sequences**

| **Primer Name** | **Primer sequence (5’ to 3’)** |
| --- | --- |
| AN10F | CGTCACTGTAGACTCACGCG |
| AN11R | CCTCTTCCCATATCTCTCTCTCTTCAAAATC |
| AN12R | CCCCTTCCCATATCTCTCTCTCTTCAAAATC |
| AN13F | GAGAGAGAGATATGGGAAGAGGTAGGGTTC |
| AN14F | GAGAGAGAGATATGGGAAGGGGTAGGGTTC |
| AN15R | CCGGATCCAAGTTCACGTTCATTC |
| AN19F | GAAAAAAGGTTACAAACGCATTAC |
| AN20R | ATCTCTCTCTCTTCAAAATCTCAAG |
| AN21F | GGAATTCATGTATCGTTTCAA |
| AN22R | CCTCTTCCCATTTTTGATCCTTTTTTAAG |
| AN23R | CCCCTTCCCATTTTTGATCCTTTTTTAAG |
| AN24F | AAGGATCAAAAATGGGAAGAGGTAGGGTTC |
| AN25F | AAGGATCAAAAATGGGAAGGGGTAGGGTTC |
| AN185F | CTTGGCTCCTTCGCCGCATGAAGC |
| AN186R | GCTTCATGCGGCGAAGGAGCCAAG |
| AN210F | GTTCGGTCGGCATCTACTCTATTC |
| AN211R | TCGGCTCCAACAATGTCCTGAC |
| AN215R | TTTTCTAGTGCGGATGTGCTT |
| AN221F | GATGGATCCTCCAATCCAGACACTGTA |
| AN222R | GTATTGTGTTGGACTCTGGTGATGGTGT |
| AN233F | CACCATGGGAAGAGGGAGAGTAG |
| AN234R | TCAAATAGAGTTGGTGTCATAAG |
| AN235F | CACCATGGGAAGAGGGAGAGTGG |
| AN237F | CACCACGGCGTACCAATCGGAGCTAG |
| AN238R | TTACACTAACTGGAGAGCGG |
| AN239F | CACCATGGCGAGAGAAAAGATTC |
| AN240R | CTAACCACCATACGGTAAGC |
| AN241F | CACCATGGGAAGAGGGAAAGTTG |
| AN242R | TCAAATGACAAATGGTTTCAG |
| AN243F | CACCATGGGAAGGGGTAGGGTTC |
| AN244R | TCATGCGGCGAAGCAGCCAAG |
| AN245R | TCATGCGGCGAAGGAGCCAAG |
| AN246F | CACCATGGGAAGAGGTAGGGTTC |
| AN247R | CTACTCGTTCGTAGTGGTAG |
| AN248R | GTTGTAAACGGGCATATTGTGGCCTTGGTTC |
| AN249F | GCCACAATATGCCCGTTTACAACTGCAAC |
| AN299F | GAGCTCTTCTTTATATCTCTCTTGTAG |
| AN301R | CATGCAAGAGTCGGTGGAAT |
| AN303F | CACCGAGCTCGAAAAAAGGTTACAAACGCATTACA |
| AN304F | CACCCGGGGAAAAAAGGTTACAAACGC |
| AN305F | CACCGGAATTCATGTATCGTTTCAA |
| AN306R | GGATCCTCATGAGAAATCATT |
| AN334F | TTGGAGAGAAACCAGAGG |
| AN373R | TCAAAGAACCCAACCTTGGACG |
| AN379F | GAGAGAGAGAGATACTTTGGTCATT |
| AN380R | TGAGCAAACCAGACCTTCTCT |
| AN381R | TGGGAGAAGACAACAAGAGCA |
| AN382F | GGAGAAGAAAACGGGTCAGC |
| AN383R | CGTAACATCCAAGCTGGAAGCTA |
| AN385R | GGACGTAACATCCAAGCCGA |
| AN386R | GTAGTGGTAGGACGTAACATCTG |
| GL10F | TCTTGAGATTTTGAAGAGAGAG |
| GL314F | GGTACCATGGGAAGAGGTAGGGTTCAG |
| GL315R | GGATCCTCATGAGAAATCATTACCAAGATATGA |
| GL373F | GGAATTCATGTATCGTTTCAAAACTCAGGAC |
| GL374R | GGGGTACCTTTTGATCCTTTTTTAAGAAACTTCTT |
| GL395F | GGGATTGACAGGATTGCCTA |
| GL418F | TGTCTGTTGTGCCCAGTCAT |
| GL419R | GCGTTCAAAAGTCGCCTAAG |
| GL768F | CAAACTCTTAGCTTCCGGCTTG |
| GL769R | GGAAGCTAAGAGTTTGGTTCC |
| GL1090F | ATGGGAAGGGGTAGGGTTC |
| GL1091R | AAGTTCACGTTCATTCTCTCTGAC |
| GL1235F | AACTCTCTGCTTCCGGCTCAGATGTTACGTCC |
| GL1236R | GGACGTAACATCTGAGCCGGAAGCAGAGAGTT |
| GL1237F | AACTCTCTGCTTTCGGCTTGGATGTTACGTCC |
| GL1238R | GGACGTAACATCCAAGCCGAAAGCAGAGAGTT |
| GL1419R | GGATCCAAGTTCACGTTCATTCTCTCTGAC |
