# Supplementary figures and images for: Functional Divergence of APETALA1 and FRUITFULL is due to Changes in both Regulation and Coding Sequence
Source: Front Plant Sci. 2015 Dec 2;6:1076. doi: 10.3389/fpls.2015.01076 (PMC4667048; doi:10.3389/fpls.2015.01076)

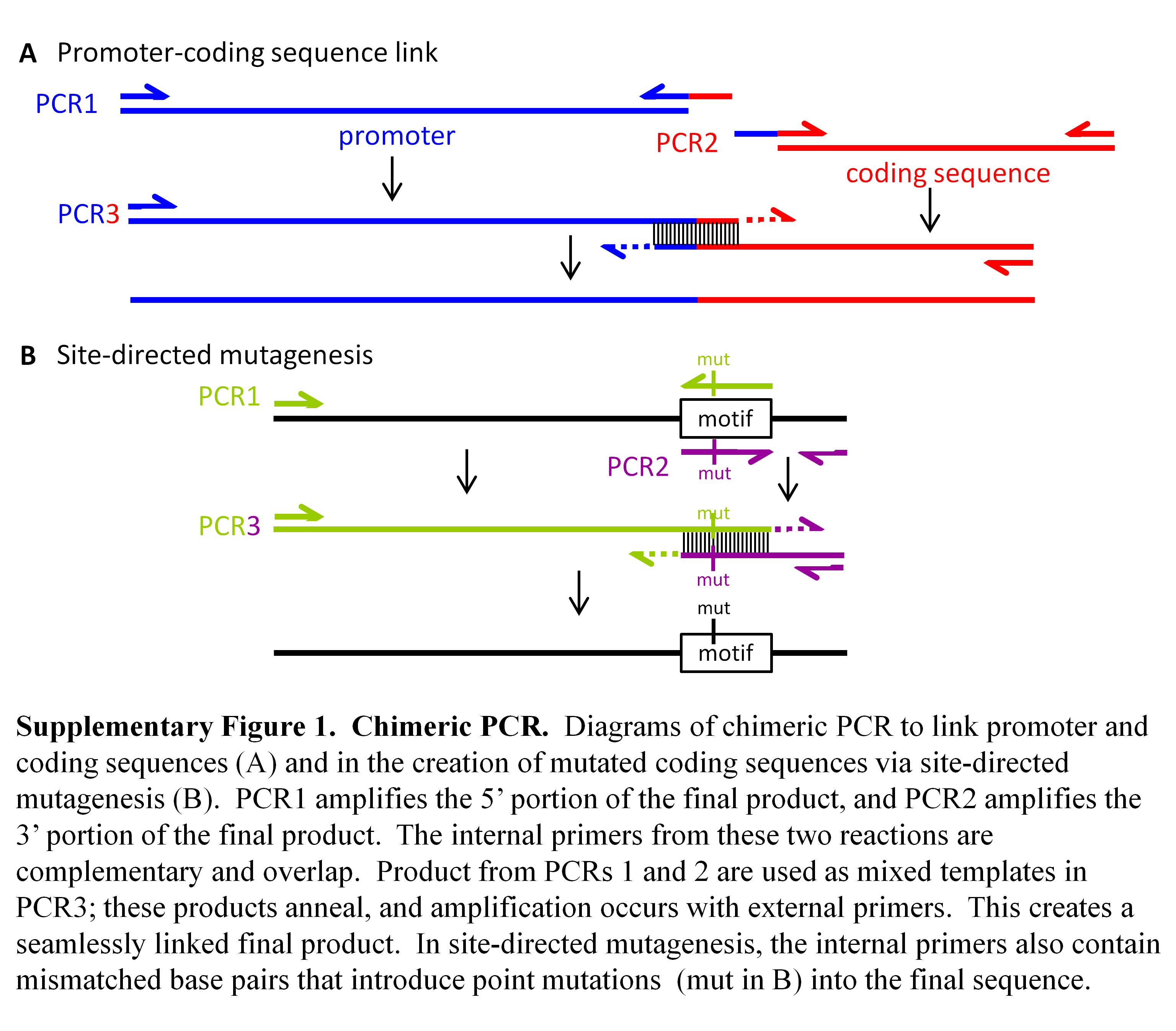

Supplement: Supplementary file 3 [file Image_1.TIF]

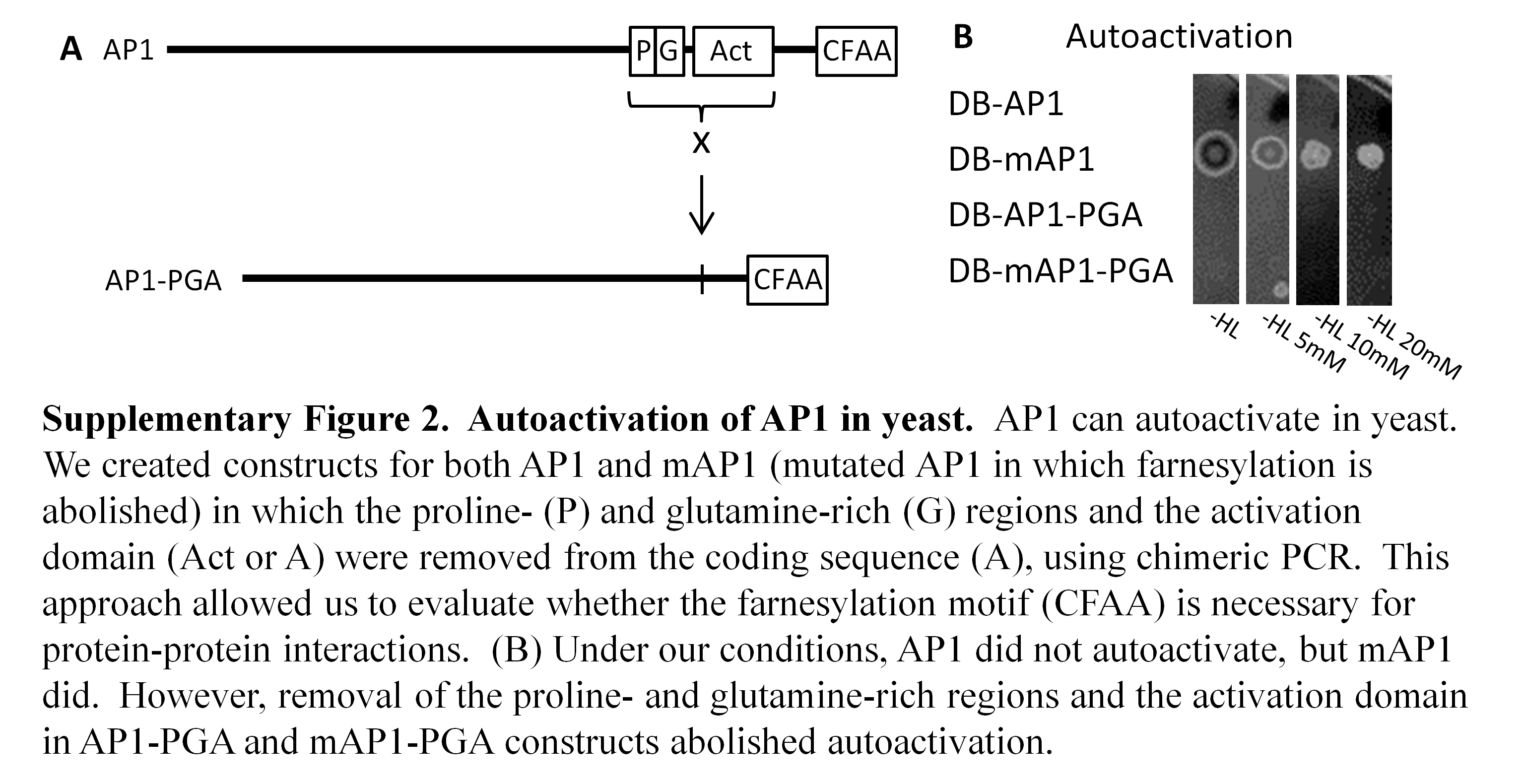

Supplement: Supplementary file 4 [file Image_2.TIF]

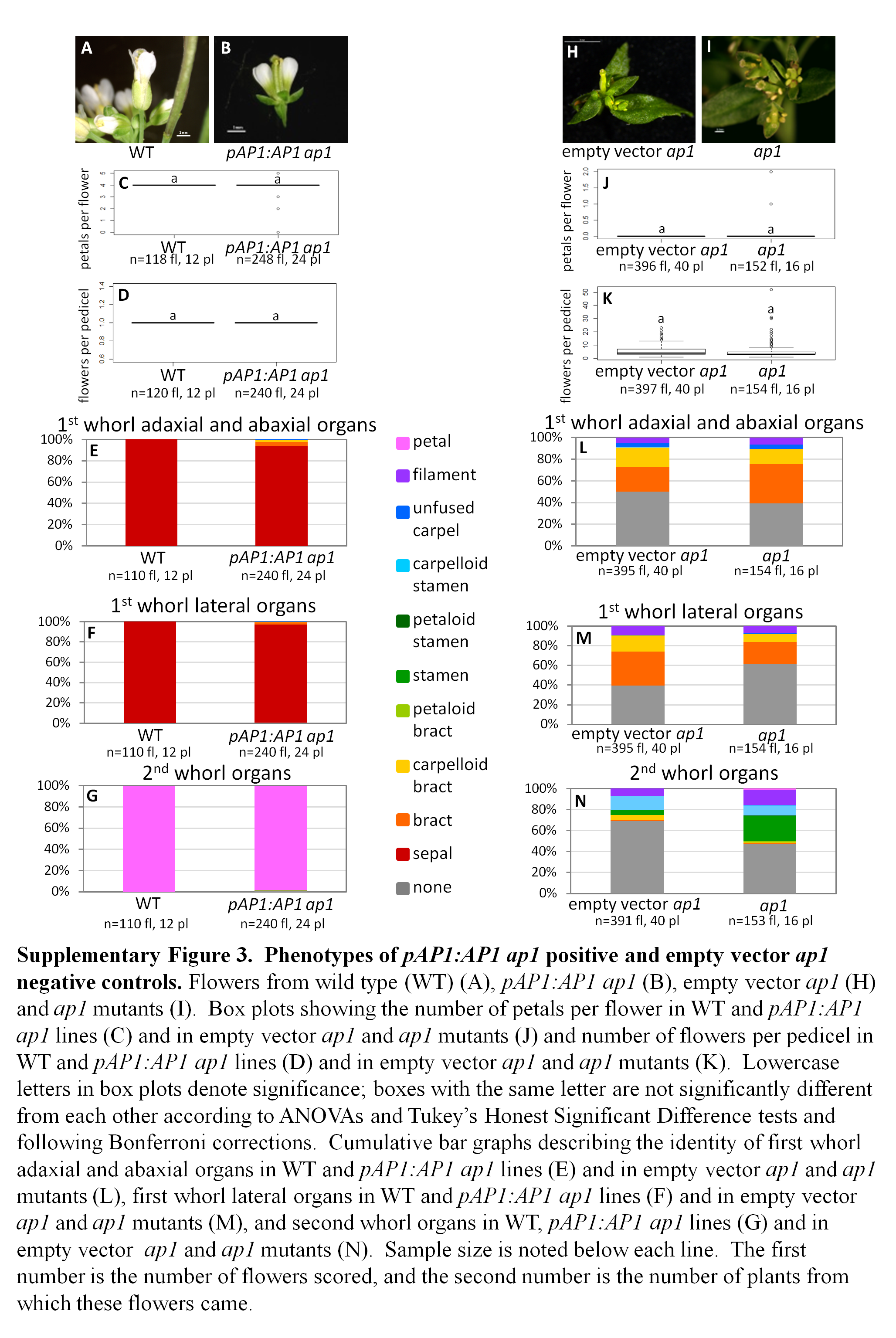

Supplement: Supplementary file 5 [file Image_3.TIF]

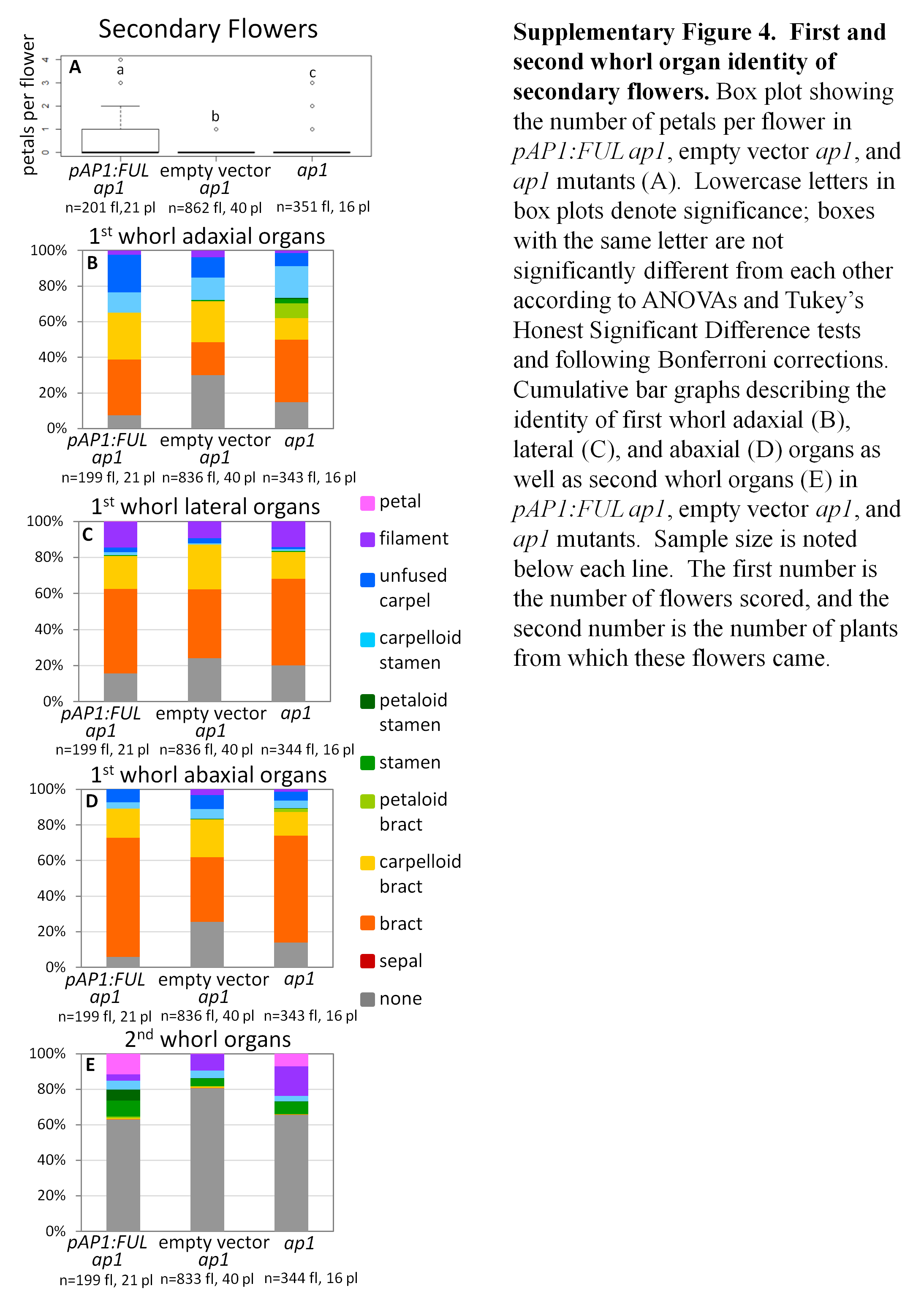

Supplement: Supplementary file 6 [file Image_4.TIF]

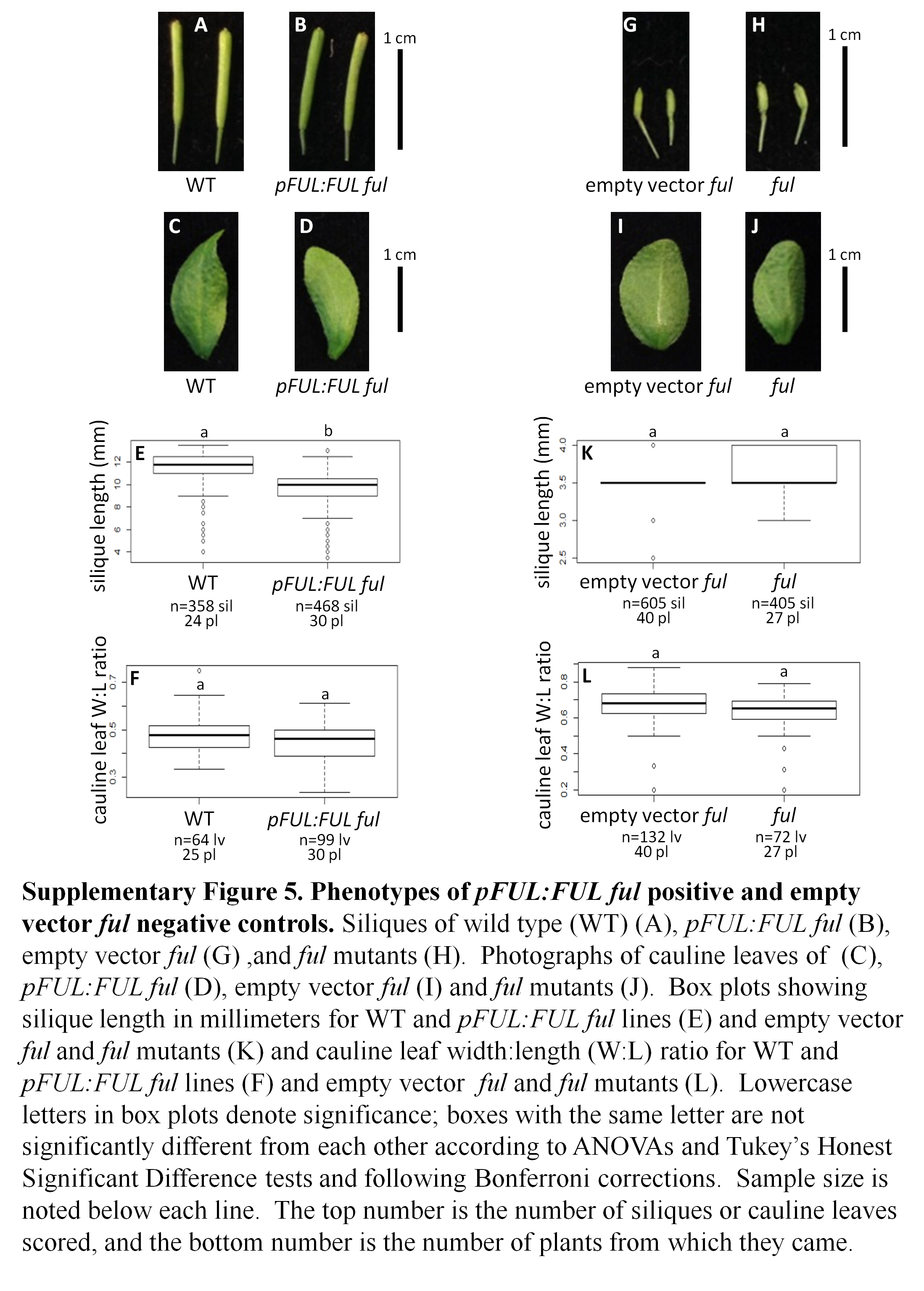

Supplement: Supplementary file 7 [file Image_5.TIF]
